# MULGA, a unified multi-view graph autoencoder-based approach for identifying drug–protein interaction and drug repositioning

**DOI:** 10.1093/bioinformatics/btad524

**Published:** 2023-08-23

**Authors:** Jiani Ma, Chen Li, Yiwen Zhang, Zhikang Wang, Shanshan Li, Yuming Guo, Lin Zhang, Hui Liu, Xin Gao, Jiangning Song

**Affiliations:** School of Information and Control Engineering, China University of Mining and Technology, Xuzhou 221116, China; Monash Biomedicine Discovery Institute and Department of Biochemistry and Molecular Biology, Monash University, Melbourne, VIC 3800, Australia; Climate, Air Quality Research Unit, School of Public Health and Preventive Medicine, Monash University, Melbourne, VIC 3004, Australia; Monash Biomedicine Discovery Institute and Department of Biochemistry and Molecular Biology, Monash University, Melbourne, VIC 3800, Australia; Climate, Air Quality Research Unit, School of Public Health and Preventive Medicine, Monash University, Melbourne, VIC 3004, Australia; Climate, Air Quality Research Unit, School of Public Health and Preventive Medicine, Monash University, Melbourne, VIC 3004, Australia; School of Information and Control Engineering, China University of Mining and Technology, Xuzhou 221116, China; School of Information and Control Engineering, China University of Mining and Technology, Xuzhou 221116, China; KAUST Computational Bioscience Research Center (CBRC), King Abdullah University of Science and Technology (KAUST), Thuwal 23955, Saudi Arabia; Monash Biomedicine Discovery Institute and Department of Biochemistry and Molecular Biology, Monash University, Melbourne, VIC 3800, Australia; Wenzhou Medical University-Monash Biomedicine Discovery Institute (BDI) Alliance in Clinical and Experimental Biomedicine, Wenzhou 325035, China; Monash Data Futures Institute, Monash University, Melbourne, VIC 3800, Australia

## Abstract

**Motivation:**

Identifying drug–protein interactions (DPIs) is a critical step in drug repositioning, which allows reuse of approved drugs that may be effective for treating a different disease and thereby alleviates the challenges of new drug development. Despite the fact that a great variety of computational approaches for DPI prediction have been proposed, key challenges, such as extendable and unbiased similarity calculation, heterogeneous information utilization, and reliable negative sample selection, remain to be addressed.

**Results:**

To address these issues, we propose a novel, unified multi-view graph autoencoder framework, termed MULGA, for both DPI and drug repositioning predictions. MULGA is featured by: (i) a multi-view learning technique to effectively learn authentic drug affinity and target affinity matrices; (ii) a graph autoencoder to infer missing DPI interactions; and (iii) a new “guilty-by-association”-based negative sampling approach for selecting highly reliable non-DPIs. Benchmark experiments demonstrate that MULGA outperforms state-of-the-art methods in DPI prediction and the ablation studies verify the effectiveness of each proposed component. Importantly, we highlight the top drugs shortlisted by MULGA that target the spike glycoprotein of severe acute respiratory syndrome coronavirus 2 (SAR-CoV-2), offering additional insights into and potentially useful treatment option for COVID-19. Together with the availability of datasets and source codes, we envision that MULGA can be explored as a useful tool for DPI prediction and drug repositioning.

**Availability and implementation:**

MULGA is publicly available for academic purposes at https://github.com/jianiM/MULGA/.

## 1 Introduction

Drug repositioning aims to explore novel clinical applications of the approved compounds for treating different diseases and can thereby save much effort and time for new drug development (Pushpakom *et al.* 2019). Repositioning existing drugs necessitates accurate identification of drugs’ target proteins and drug–protein interactions (DPIs). Existing computational approaches can be broadly categorized into molecular docking-based and machine learning-based methods. Molecular docking (Salsbury 2010) is a traditional but important approach for predicting drug–protein binding affinities, which highly relies on the availability of 3D structures of proteins and drugs. As such, the practical application of molecular docking is limited when the 3D structures of drugs, proteins, and their physiochemical data still need to be improved. On the other hand, machine learning-based approaches construct various predictive models, such as matrix completion-based and deep learning (DL) models, using curated DPI training data. Matrix factorization and subspace learning methods have been extensively applied to DPI prediction due to their adaptability in integrating prior information. Drug similarity and protein similarity matrices are indispensable prior knowledge in the matrix completion-based DPI prediction methods, such as NRLMF (Liu *et al.* 2016) and SPLCMF (Xia *et al.* 2019), given the assumption that similar drugs tend to target similar proteins. However, obtaining an extendable and unbiased similarity matrix for proteins/drugs is challenging due to the large number of molecular and versatile physiochemical features of drugs/proteins and various similarity measures.

In recent years, DL techniques have attracted considerable attention and demonstrated an emerging promise in DPI prediction due to its flexible architecture and capability of capturing useful latent features. Two primary types of DL-based approaches have been proposed for DPI prediction, including Natural Language Processing (NLP)-based DL models and Graph Neural Network (GNN)-based models. NLP-based DL models, such as DeepDTA (Ozturk *et al.* 2018), DeepCDA (Abbasi *et al.* 2020), and HyperAttentionDTI (Zhao *et al.* 2022), processed protein and drug SMILES (simplified molecular-input line-entry system) sequences as sentences via the NLP algorithms for extracting semantic features. However, there are two common limitations in NLP-based DL methods: (i) the syntax of the drug SMILES sequence and protein sequence is different from the natural language, which inevitably results in the omission of key characteristics during the feature learning process; and (ii) the structural and physiochemical properties of drugs and proteins are ignored by the language models. GNN-based models, such as GraphBAR (Son and Kim 2021), GraphDTA (Thin *et al.* 2021), and AttentionSiteDTI (Yazdani-Jahromi *et al.* 2022), were then proposed to naturally model the 3D structure of drug–target complex or drug molecules to extract the structural features of drugs and targets systematically. GraphBAR modeled the 3D structure of each drug–target binding complex as a graph, in which the nodes are the atoms of drugs and proteins within a fixed cutoff distance threshold in the binding complex. Coupled with the attention pooling mechanism, AttentionSiteDTI used the graph attention embedding networks to extract learnable feature embeddings from the graph representations of drug’s SMILES and proteins’ binding sites. However, a drug–protein binding complex often necessitates multiple large-scale, high-dimension, and sparse matrices to represent its entire structures, resulting in redundant input to the model, which will possibly lead to higher computational costs and a reduction of the model performance. To address these issues, DrugVQA (Zheng *et al.* 2020) used the contact map (CMAP) to present sequence-derived and structural features. However, it is essential to mention that CMAP also presents the protein structure prediction results, which may need to be sufficiently reliable for further prediction and analyses.

In this study, we proposed a unified Multi-view Learning and Graph Autoencoder framework, termed MULGA, for DPI prediction and drug repositioning. By leveraging a multi-view learning module and a graph autoencoder module, MULGA provides important insights into molecular similarities in a metric-free manner and extrapolates the topological characteristics and the correlation patterns embedded in the drug–protein heterogeneous network.

## 2 Materials and methods

### 2.1 Datasets

We comprehensively evaluated our method on four benchmark datasets, including one extracted from DrugBank (released on 2022.01.04; version 5.1.9) (Wishart *et al.* 2018), and the other three extracted from the Kinase Inhibitor Bioactivity (KIBA) dataset (Tang *et al.* 2014), Davis Kinase Inhibitor (Davis, version 0.3.2) dataset (Davis *et al.* 2011), and BindingDB dataset (Liu *et al.* 2007), respectively. These four benchmark datasets are introduced in detail in the following subsections.

#### 2.1.1 DrugBank

To ensure the quality of the dataset, we manually discarded those drugs whose sequences in the Simplified Molecular-Input Line-Entry System (SMILES) format were not recognizable according to RDKit (Landrum 2019).To address the sparsity issue of the DPI dataset, we further retained the drugs that target at least two proteins. The sparsity was calculated as the number of unknown DPIs divided by that of all collected DPIs. The final dataset contained 6871 validated DPIs with a sparsity of 99.71%, including 1822 drugs and 1447 proteins, respectively.

#### 2.1.2 KIBA

KIBA is a valuable benchmark dataset for DPI prediction. It includes comprehensive bioactivity measurements (e.g. *K*(d), *K*(i) and IC50) of 2111 kinase inhibitors against 229 kinases. Specifically, DPI pairs that have *K*(d) values <30 units are considered as binding. After downloading and filtering using the Therapeutics Data Commons (TDC) tool, a drug discovery application developed by Huang *et al.* (2022), we obtained 22 154 binarized DPIs involving 1720 drugs and 220 proteins.

#### 2.1.3 Davis

The Davis dataset consists of wetlab-tested *K*(d) values among 72 kinase inhibitors and 442 kinases, covering more than 80% of human catalytic protein kinome. After the processing with the TDC tool, 7320 positive samples were obtained with 68 drugs and 379 proteins.

#### 2.1.4 BindingDB

BindingDB serves as a public repository of experimentally measured binding affinities between small molecules (ligands) and their target biomolecules (proteins), which were collected from the literature and public datasets. It consists of *K*d values among 10 665 drugs and 1413 proteins. Furthermore, 9166 DPIs among 3400 drugs and 886 proteins were retained after being binarized and filtered with the TDC tool.

As for each dataset, based on the SMILES sequences of drugs and UniProt IDs of proteins, we extracted Molecular Access System (MACCS) keys fingerprint (Durant *et al.* 2002), topological fingerprint (Carhart *et al.* 1985), and Morgan fingerprint (Morgan 1965) using the RDkit package. To systemically describe protein sequences and physiochemical properties, amino acid composition (AAC) (Morgan 1965), Moran autocorrelation (Feng and Zhang 2000), Composition/Transition/Distribution (CTD) (Cai *et al.* 2004), and pseudo-amino acid composition (PAAC) (Chou 2005) were calculated using the *iLearn* software package (Chen *et al.* 2020, 2021). The curated DrugBank, KIBA, Davis, and BindingDB datasets and the feature extraction pipeline are accessible at https://github.com/jianiM/MULGA/.

### 2.2 Overview of the MULGA framework

**Figure 1.**
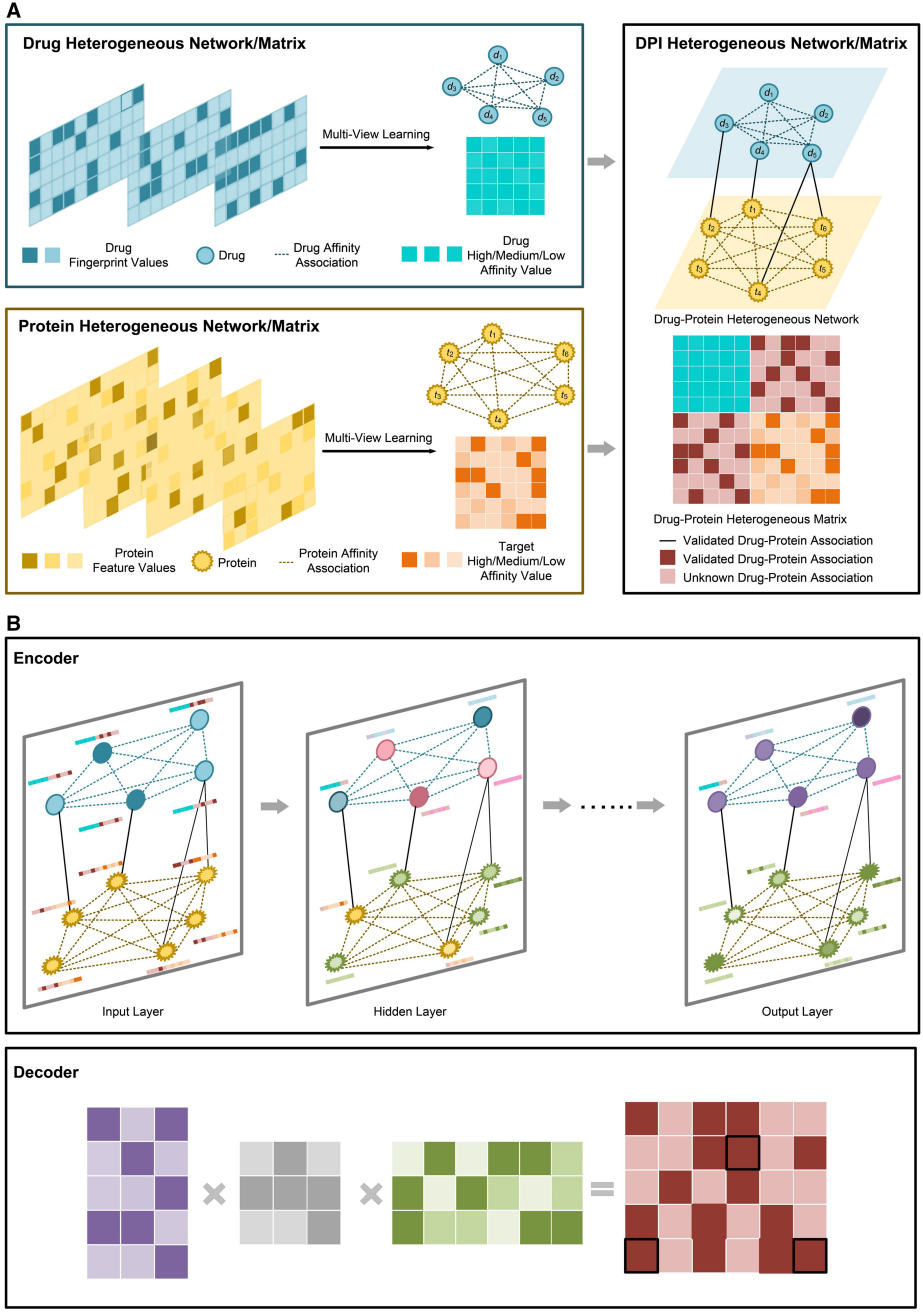
A schematic workflow of the MULGA methodology. The pipeline consists of two main parts: (A) affinity matrix learning and drug–protein heterogeneous matrix construction. MULGA applies the multi-view learning strategy to multiple drug fingerprints and multiple protein features to generate a drug affinity matrix and protein affinity matrix in a feature-driven way. By incorporating the affinity matrices and known DPI associations, a drug–protein heterogeneous graph is constructed. (B) DPI inference with the graph autoencoder. Taking the drug–protein heterogeneous graph as the input, MULGA uses a graph autoencoder to effectively learn the node feature embeddings and topological patterns of the input graph, and then infers the unknown DPIs using the encoder–decoder scheme.

MULGA consists of three major steps, including (i) *feature-driven affinity matrix learning*, (ii) *DPI inference*, and (iii) *“guilty-by-association”-inspired negative sampling*. Accurate and unbiased molecular affinity matrices play vital roles in DPI prediction. At the first step, MULGA leverages a multi-view technique to extract the complementary information embedded in the protein and drug features and obtain protein and drug affinity matrices in a similarity metric-free manner. A drug–protein heterogeneous network is then spontaneously constructed by incorporating known drug–protein associations and drug and protein affinity matrices. In the second step, a graph autoencoder using the GCN as the encoder is constructed to learn the low-dimensional representations of drugs and proteins to explore the topological information embedded in the heterogeneous drug–protein network. The graph autoencoder then applies a matrix tri-factorization-inspired formula as a decoder to reconstruct the DPIs. In the last step, a “*guilty-by-association*”-based negative sampling method is employed to select the reliable negative samples (i.e. non-DPIs) based on the assumption that similar drugs target similar proteins. The overall workflow of our proposed approach is illustrated in Fig. 1.

#### 2.2.1 Leveraging multi-view learning to enable effective feature-driven affinity matrix learning

To date, the mainstream approaches for DPI prediction are similarity-based, which deployed different metrics using various drug and protein features, such as the Jaccard similarity coefficient for binary features and the Cosine similarity coefficient for continuous features, respectively. Features extracted from different perspectives present a diverse landscape of drug and protein molecules. It is, therefore, crucial to efficiently incorporate these heterogeneous molecule features that represent different views of the molecules for inferencing DPIs. Based on the assumption that diverse drug/protein features originate from the common underlying latent drug/protein space, multi-view learning can capture consistent and complementary information by seeking a common subspace representation, that is, the drug affinity matrix and protein affinity matrix, across all views. Here, we leveraged multi-view learning techniques in our drug repositioning task. Specifically, we applied the pairwise multi-view learning approach (Brbic and Kopriva 2018), which builds an “agreement” matrix by using the affinity matrix of each view and balancing the trade-off between the low-rank and sparsity property constraints on the affinity matrix. The overall pipeline of the multi-view-based affinity matrix learning is illustrated in Fig. 1A.

We then elucidate the pairwise multi-view learning model used in this work. Let $n_{d}$ and $n_{p}$ denote the numbers of drugs and proteins respectively. For simplicity, $n_{d}$ and $n_{p}$ are collectively denoted as n, while the number of drug features and protein features are collectively denoted as v. The pairwise multi-view learning model is shown as (1):

(1) $$\underset{A^{\left(1\right)},A^{\left(2\right)},\ldots ,A^{\left(v\right)}}{\min}\sum_{i=1}^{v}\left(\beta ||A^{\left(i\right)}|{|}_{*}+(1-\beta )||A^{\left(i\right)}|{|}_{1}\right)+\sum_{\begin{array}{c} 1\leq i,j\leq v\\ i\neq j \end{array}}||A^{\left(i\right)}-A^{\left(j\right)}|{|}_{F}^{2}s.t.\;X^{\left(i\right)}=X^{\left(i\right)}A^{\left(i\right)},\mathrm{diag}(A^{\left(i\right)})=0,i=1,\ldots ,v,$$

where $X^{\left(i\right)}\in R^{d\times n}$ denotes the *i*-th drug/protein feature, d represents the dimension of the *i*-th feature, $A^{\left(i\right)}\in R^{n\times n}$ demonstrates the drug/protein affinity matrix for view i, while ${\left||\;|\right|}_{*}$ and ${\left||\;|\right|}_{1}$ are the nuclear norm and the L_1_-norm, respectively. ${\left||\;|\right|}_{*}$ is to guarantee the low-rank property of affinity matrix $A^{\left(i\right)}$, while ${\left||\;|\right|}_{1}\;$keeps the sparsity property of $A^{\left(i\right)}$. *β* is introduced to balance the trade-off between the low-rank property and the sparsity of every affinity matrix. Equipped with low-rank and sparse constraints, pairwise multi-view learning efficiently reduces the redundancy of the diversity of the molecular features and simultaneously extracts complementary information across various views. $\sum_{1\leq i,\;j\leq v,\;i\neq j}{\left||A^{\left(i\right)}-A^{\left(j\right)}|\right|}_{F}^{2}$ is used to achieve the common consensus representation of the drug/protein affinity matrix, and $X^{\left(i\right)}=X^{\left(i\right)}A^{\left(i\right)}$ denotes that each drug can be represented as a linear combination of the other drugs from the drug space, and so as for proteins. We also used the constraint*** diag***(***A***^(^^*i*^^)^) to avoid the trivial solution of representing a drug/protein as a linear combination of itself. Multi-view learning model described in Equation (1) conveys that without any similarity metrics, the view-specific affinity matrices ***A***^(1)^, ***A***^(2)^,…, ***A***^(^^*v*^^)^ could be derived in an automatically feature-driven manner. The updating rule for each $A^{\left(i\right)}$ (i.e. for views ***A***^(1)^, ***A***^(2)^,…, ***A***^(^^*v*^^)^) at the *k*-th iteration has been listed in the Supplementary File.

Next, a series of affinity matrices of all views, i.e. ***A***^(1)^, ***A***^(2)^, ***…***, ***A***^(^^*v*^^)^, can be obtained. Subsequently, we used three element-wise operators (e.g. max, min and average) to generate the joint affinity matrix ***A***. By deploying pairwise multi-view learning on multiple drug and protein features, we can obtain the joint drug affinity matrix ${\;A}_{\mathrm{DD}}{\;\in R}^{n_{d}\times n_{d}}$ and the joint protein affinity matrix $A_{\mathrm{PP}}{\;\in R}^{n_{p}\times n_{p}}$. Then we applied the min-max normalization approach on $A_{\mathrm{DD}}$ and $A_{\mathrm{PP}}$, respectively. In MULGA, the DPI matrix is denoted as a binary matrix $A_{\mathrm{DP}}{\;\in R}^{n_{d}\times n_{p}}$, where the validated DPIs are denoted as “1”, or “0” vice versa. Incorporating ***A_DP_***, ***A_DD_***, and ***A_PP_***, we built a heterogeneous drug–protein graph, where the nodes denote drugs or proteins, and the edges connecting two nodes denote the similarity between two drugs or proteins or the validated associations between them. The adjacency matrix of the heterogeneous drug–protein graph is denoted as ***H***, where ***A_PD_*** is the transpose of ***A_DP_***:

(2) $$H=\left(\begin{array}{cc} A_{DD} & A_{DP}\\ A_{PD} & A_{PP} \end{array}\right)\in R^{\left(n_{d}+n_{p})\times (n_{d}+n_{p}\right)}.$$

***H*** is a symmetric matrix, which guarantees proper properties for downstream matrix calculation and can especially enhance computational efficiency. Simultaneously, it also substantially reduces the sparsity when compared to using only the association matrix ***A_DP_***.

#### 2.2.2 DPI inference with graph autoencoder

In MUGLA, we inferred unknown DPIs using a graph autoencoder. As shown in Fig. 1B, the encoder consists of multiple GCN layers, while the decoder is implemented by the inner-product operation. During this process, the quality of the embedding determines the quality of the reconstructed output. Therefore, if the lower dimensional embedding captures more information and learns more accurate embeddings from the input, the model performance can be significantly improved. Simultaneously, the heterogeneous drug–protein network is a graph, rather than an image or sentence, which contains the structural information the other two do not. Thereby, GCN comes naturally as an encoder to learn its embedding features. Taking the heterogeneous drug–protein graph as an input, graph autoencoder learns the compressed drug/protein embeddings in a “message-passing” way by aggregating the information from their neighbors without changing the graph structures (Kipf 2017). Mathematically, the layer-wise propagation rule in a graph autoencoder can be defined as (3):

(3) $$H^{\left(l+1\right)}=\sigma (D^{-\frac{1}{2}}GD^{-\frac{1}{2}}H^{\left(l\right)}W^{\left(l\right)}),$$

where $D^{-\frac{1}{2}}GD^{-\frac{1}{2}}$ denotes the normalized graph Laplacian, ***G*** is the adjacency matrix of input graph, $D=\mathrm{diag}(\sum_{j}G_{ij})$ represents the degree matrix, ***H***^(^^*l*^^)^ is a feature embedding matrix, ***W***^(^^*l*^^)^ is a learnable weight matrix, and σ denotes an activation function. Here, to efficiently filter the useful information of the heterogeneous graph, we set the affinity threshold τ as 0.80. Based on the following:

$${\left({\hat{A}}_{DD}\right)}_{ij}=\left\{ \begin{array}{c} 1,\;\text{if}\;\;(A_{DD}{)}_{ij}\geq \tau \\ 0,\;\text{if}\;\;(A_{DD}{)}_{ij}<\tau \end{array}\right.,\;\;{\left({\hat{A}}_{PP}\right)}_{ij}=\left\{ \begin{array}{c} 1,\;\text{if}\;\;(A_{PP}{)}_{ij}\geq \tau \\ 0,\;\text{if}\;\;(A_{PP}{)}_{ij}<\tau \end{array}\right.,$$

via which we obtain

(4) $$\hat{H}=\left(\begin{array}{cc} {\hat{A}}_{DD} & A_{DP}\\ A_{PD} & {\hat{A}}_{PP} \end{array}\right)\in R^{\left(n_{d}+n_{p})\times (n_{d}+n_{p}\right)}.$$

The first layer receives***H*** as input features and $\hat{H}$ as the adjacency matrix of the input graph, respectively (i.e. ***H***^(0)^ = ***H*** and ***G*** = $\hat{H}$). The multiplication of$\;D^{-\frac{1}{2}}GD^{-\frac{1}{2}}H^{\left(l\right)}$, termed Laplacian smoothing, aggregates and averages the first-order neighbor information including the affinity patterns and association patterns for each node. Then the result of Laplacian smoothing is transformed by the weight matrix ***W***^(^^*l*^^)^ and activation function σ. It is important to note that the learnable weight matrix in the first layer was set as$\;W^{\left(0\right)}=R^{(n_{d}+n_{p})\times k}$. As the result, $H^{\left(1\right)}=\sigma (D^{-\frac{1}{2}}GD^{-\frac{1}{2}}H^{\left(0\right)}W^{\left(0\right)})\in R^{(n_{d}+n_{p})\times k}$. Then we set $W^{\left(l\right)}=R^{k\times k},l=1,\ldots ,L$ in the subsequent layer. The stacking of multiple graph convolutional layers increases the flow of the message. Additionally, the higher layer can capture multi-hop neighboring information. In the end, MUGLA learns the graph embedding $H^{\left(L\right)}$ for drugs and proteins, where H(L)=HDHP∈R(nd+np)×k, $H_{D}\in R^{n_{d}\times k}$ is the drug embedding and $H_{P}\in R^{n_{P}\times k}$ is the protein embedding. Additionally, the learnable weight matrix in the final layer ${\;W}^{\left(L\right)}$ measures the map from drug space to the protein space. Inspired by matrix tri-factorization, MUGLA introduced an inner-product encoder to reconstruct the DPI matrix:

(5) $$H^{*}=\sigma (H_{D}W^{\left(L\right)}H_{P}^{T}),$$

where the (*i*, *j*)-th element of H*, denoted as h_ij_^*^, is the predicted association score between the drug *i* and target *j*.

#### 2.2.3 “Guilty-by-association”-based negative sampling with the similarity information

**Figure 2.**
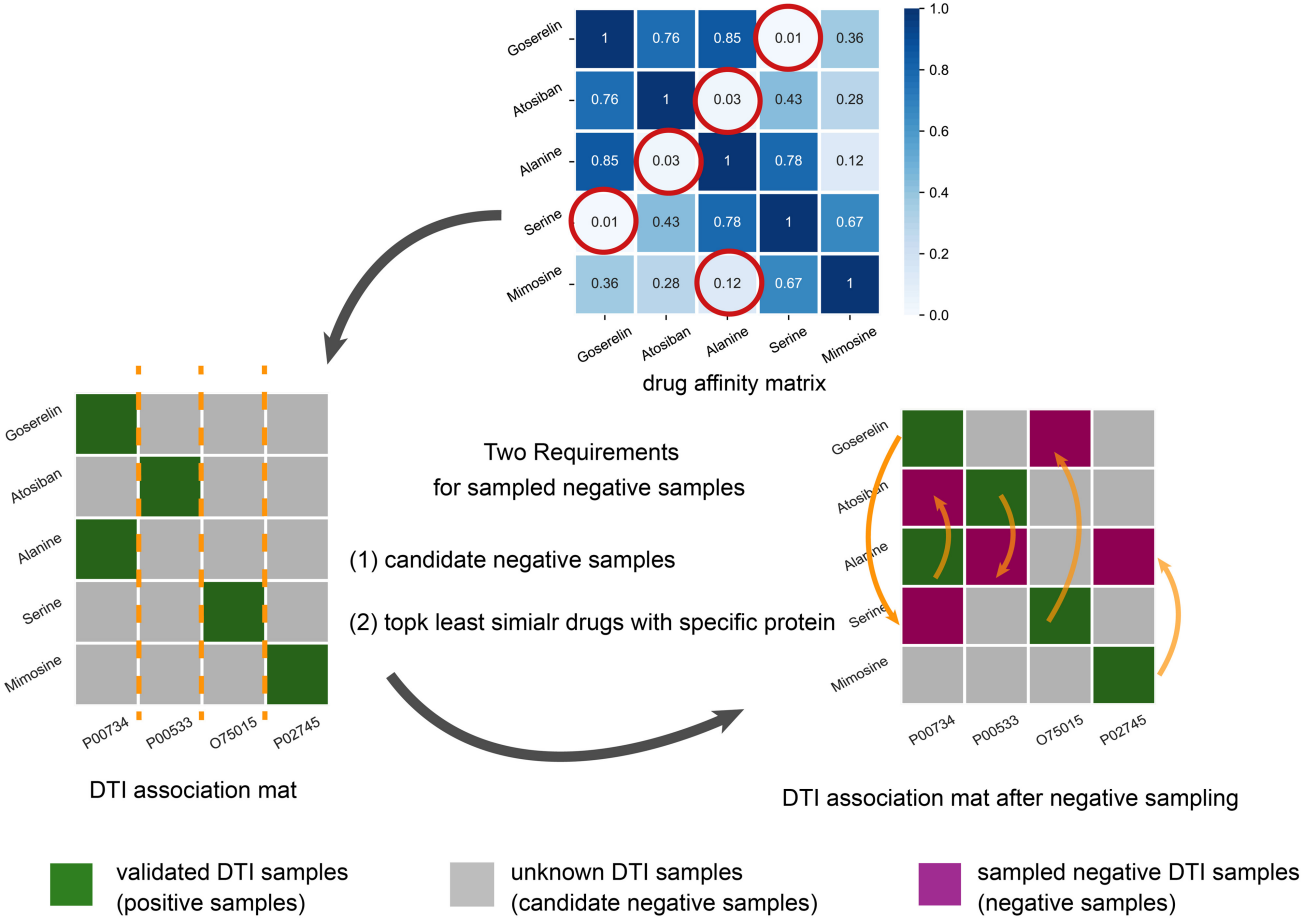
Illustration of the proposed “guilty-by-association” negative sampling scheme. Positive samples are represented by green blocks, while candidate negative samples are depicted as grey blocks. In light of the affinity scores among the selected drug and other drugs, the minimum affinity scores are circled with red, indicating the candidate drugs that are less likely to bind to the common target with the selected drugs. The sampled negative samples, obtained through the “guilty-by-association” approach, are shown as purple blocks, and the negative sampling process is protein-based, as indicated by the orange dash line, further establishing the mapping relevance of the negative samples to the existing positive samples.

A well-designed machine-learning model highly depends on the quality of the training data, wherein the negative and positive samples are equally important. Compared to positive samples (i.e. annotated DPIs), negative samples (i.e. non-associated drug–protein pairs) are difficult to identify, posing significant challenges of testing the performance of machine learning models to predict DPIs. Most studies either directly took unlabeled DPIs as the negative samples or randomly sampled the negative DPIs from the unlabeled DPIs (Thin *et al.* 2021, Zhao *et al.* 2022). This will introduce data noise to distinguish the positives from negatives, thereby resulting in unsatisfactory performance of DPI prediction. To overcome this issue, we proposed a “guilty-by-association”-based negative sampling method to select reliable negative samples, and Fig. 2 illustrates its sampling process. The “guilty-by-association” paradigm indicates that if the majority of a drug’s “neighbors” (i.e. drugs with higher similarity scores) can interact with a protein, then the drug is likely to bind to that target. This notation aligns well with the observation that the drugs with similar bioactivities can bind to a shared target, even if their structures differ. Accordingly, drug’s “neighbors” that are less similar to the compared drug are less likely to bind to the common target.

## 3 Results

### 3.1 Experimental design

To systematically evaluate the capability of MULGA in drug repositioning, we implemented MULGA in two experimental scenarios, including the “warm-start” and “cold-start-for-protein” scenarios on both two benchmark datasets. Particularly, in the “warm-start” scenario, we evaluated MULGA using both balanced (i.e. #positive samples:#negative samples = 1:1) and imbalanced (#positive samples:#negative samples = 1:10) datasets via stratified 10-fold cross-validation test. In each fold, the percentage of each class is identical. The performance was evaluated in terms of the area under the receiver-operating characteristic curve (AUROC) and the area under the Precision–Recall curve (AUPR) considering that AUPR is more informative than AUROC when testing on the imbalanced data (Fawcett, 2006, Saito and Rehmsmeier 2015), followed by other quantitative measurements in terms of ACC, Precision, Recall, Specificity, and F1 score. The corresponding performance results are provided in the Supplementary File.

Cold start poses a challenge and obstacle in drug repositioning (Nguyen *et al.* 2022). Since most proteins lack the interaction knowledge with drugs, we performed the “cold-start-for-protein” experiment to reposition the existing drugs as therapeutic targets of novel proteins. Briefly, each protein can be regarded as a novel target by removing all its associated drugs. In this way, the removed proteins and their associated drugs are regarded as test samples, while the remaining DPIs are treated as training samples. Afterwards, by applying the “cold-start-for-protein” scheme to each target, we obtained 1447 AUROC values for the DrugBank dataset, 220 for KIBA, 379 for Davis, and 886 for BindingDB, respectively. Without the prior DPI association information of the novel proteins, the “cold-start-for-protein” strategy may incur catastrophic drop in the predictive performance. Nevertheless, it is more practical for the drug repositioning problem when compared with the “warm-start” scenario. The implementation details and hyperparameter tuning procedures are provided in the Supplementary File.

### 3.2 Benchmarking MULGA with state-of-the-art methods for DPI prediction in both the “warm-start” and “cold-start-for-protein” scenarios

In this section, to comprehensively evaluate the predictive performance of MULGA, we benchmarked the optimized MULGA model with six state-of-the-art methods, including DeepConv-DTI (Lee *et al.* 2019), LRSpNM (Wu *et al.* 2021), GraphDTA (Thin *et al.* 2021), DeepDTA (Ozturk *et al.* 2018), HyperAttentionDTI (Zhao *et al.* 2022), and MLMC (Yan *et al.* 2022) under both the “warm-start” and “cold-start-for-target” scenarios. The detailed hyperparameter tuning and element-wise operator determination procedures can be found in the Supplementary File. A brief introduction of those competing methods is also provided in the Supplementary File.

**Figure 3.**
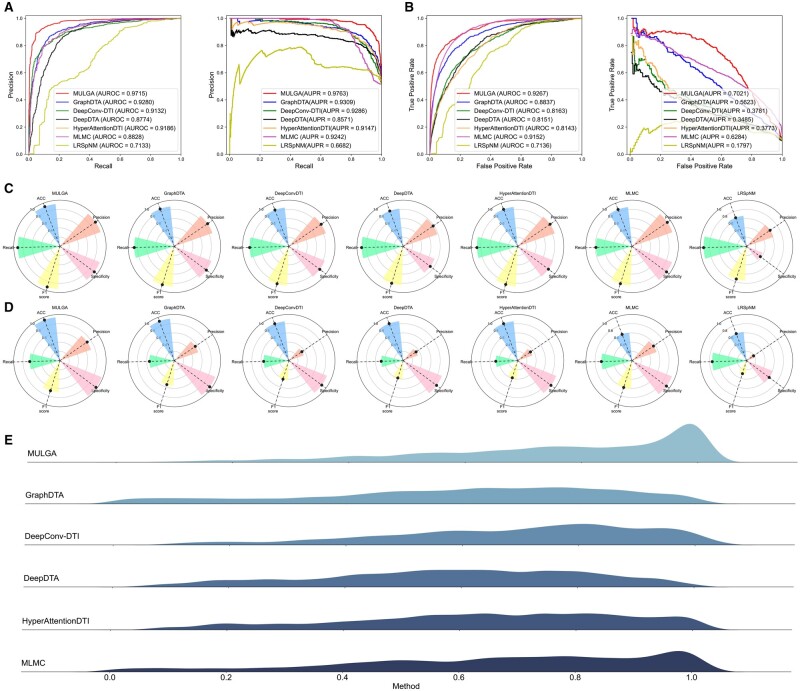
Performance comparison of MULGA and other competing methods on DrugBank dataset. (A) The ROC and PR curves of MULGA and its competing methods on the balanced dataset. (B) The ROC and PR curves of MULGA and its competing methods on the imbalanced dataset. (C) Radar plots of MULGA and other methods on ACC, Precision, Recall, F1 score, and Specificity metrics on the balanced dataset. (D) Radar plots of MULGA and other methods on ACC, Precision, Recall, F1 score, and Specificity metrics on the imbalanced dataset. (E) Performance comparison between MULGA and its competing methods in the “cold-start-for-protein” scenario.

In the “warm-start” scenario, we applied the negative sampling scheme in the training procedure for each method in order to make a fair comparison. We reported the mean value and standard variance of each method with the optimized parameter configurations on both balanced and imbalanced data settings (#postive samples:#negative samples = 1:1 for balanced data setting, and #postive samples:#negative samples = 1:10 for imbalanced data setting). The performance comparison of our method and the competing methods under both two scenarios on the DrugBank, KIBA, DAVIS, and BindingDB datasets are provided in Fig. 3 below and Supplementary Figs S2–S4, respectively.

Figure 3A and B and Supplementary Figs S2–S4A and B illustrate the performance comparison of these seven methods in terms of the ROC and PR curves on both the balanced and imbalanced settings of DrugBank, KIBA, Davis, and BindingDB, respectively. It can be observed that MULGA showed consistent superiority with the highest AUROC scores as well as the highest AUPR scores on both balanced and imbalanced “positive:negative” ratios across the datasets. In contrast, LRSpNM, a matrix completion-based method, attained the lowest performance in all experimental settings and all datasets, presumably due to the sparseness of the DPI matrix and manually calculated (thus probably) inaccurate similarity metrics. On the DrugBank dataset, MULGA outperformed the competing methods with a relatively large margin by 4.35%–25.36% in terms of AUROC and 4.54%–30.82% in terms of AUPR on the balanced setting, and by 1.07%–21.31% in terms of AUROC and 13.98%–52.24% in terms of AUPR on the imbalanced setting, respectively. Moreover, MLMC performed a bit worse than MULGA. The excellent performance of both methods may be attributed to the incorporation of multi-faceted drug/protein features, as well as the unbiased similarity information extracted by multi-view learning methods. On the KIBA, Davis, and BindingDB datasets, MULGA also achieved a large margin of improved performance on both balanced and imbalanced datasets. The performance of these methods, in terms of ACC, Recall, Precision, F1 score, and Specificity on the balanced imbalanced datasets is shown in Fig. 3C and D for the DrugBank dataset, and Supplementary Figs S2–S4C and D for the KIBA, Davis, and BindingDB datasets, respectively.

Based on the prior similarity information incorporated in our constructed drug–protein heterogeneous network, MULGA can further predict novel targets of candidate drugs in the “cold-start-for-protein” setting, which can be of practical importance in real-world applications. Due to its malfunction under the “cold-start-for-protein” setting, LRSpNM was excluded from the performance comparison. Taking the DrugBank dataset as an example, we obtained 1447 AUROC scores for each method by deploying the “cold-start-for-protein” scheme to each target. All the 1477 AUROC values achieved by MULGA, DeepDTA, GraphDTA, DeepConv-DTI, HyperAttentionDTI, and MLMC for the DrugBank dataset are shown in Fig. 3E, while for the KIBA, Davis, and BindingDB datasets, AUROC distribution of six compared methods are shown in Supplementary Figs S2–S4E, respectively. We can see from Fig. 3E that the AUROC scores exhibited right-skewed distributions with values ranging from 0.8 to 1.0, and MLMC showed a slightly right-skewed distributions with the AUROC scores distributed within the interval [0.6, 0.9]. In contrast, GraphDTA, DeepConv-DTI, and HyperAttentionDTI exhibited normal distributions with the AUROC scores ranging from 0.4 to 0.8. Combined with AUROC distributions of them generated by the KIBA, Davis, and BindingDB datasets (Supplementary Figs S2–S4E), we concluded that MULGA significantly outperformed the competing methods under the “cold-start-for protein” scenario.

### 3.3 Importance of the multi-view strategy on the predictive performance of MULGA

**Figure 4.**
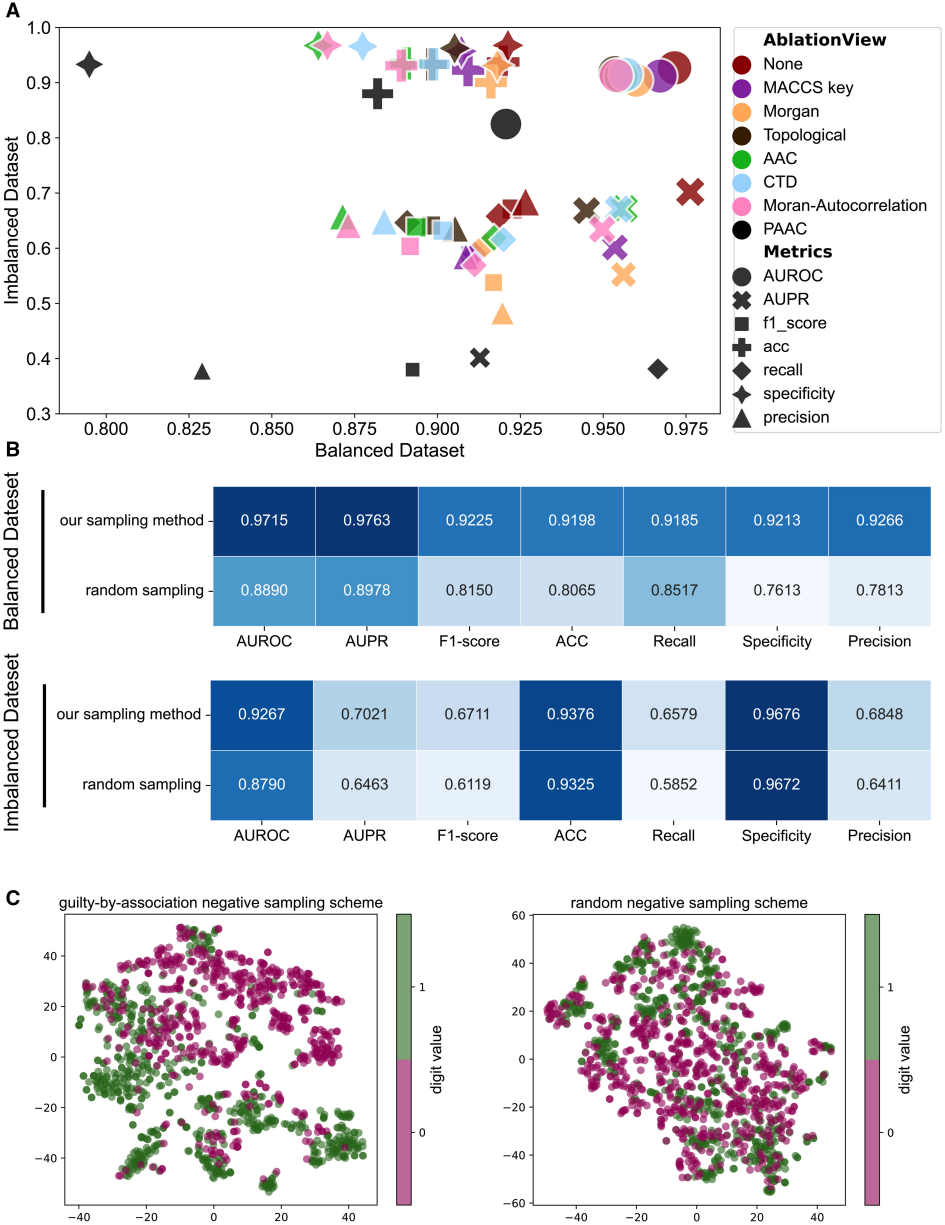
Results of view ablation studies on DrugBank dataset. (A) Performance comparison of different view ablation studies on the balanced dataset and imbalanced dataset, respectively. The larger markers correspond to larger values for each metric. (B) Performance comparison of MULGA with our proposed “guilty-by-association” negative sampling and that with random negative sampling on the balanced dataset and imbalanced dataset, respectively. Darker blue denotes a higher score while lighter blue corresponds to a lower score. (C) TSNE visualizations of the feature space distribution of MULGA with our proposed “guilty-by-association” negative sampling and the random negative sampling.

MULGA incorporates three types of drug features and four types of protein features for the DPI prediction in a multi-view manner. Each feature type represents a “view” that provides potentially useful information for portraying drugs or proteins. In this section, we performed a “view-ablation” experiment on the DrugBank dataset to evaluate the importance and effectiveness of each view. In brief, after removing each feature view, the MULGA were trained on the remaining features under the “warm-start” scenario. Figure 4A shows the performance of view-ablation experiments in terms of various measures on both balanced and imbalanced datasets.

As illustrated in Fig. 4A and Supplementary Table S2, when compared to the MULGA model trained using all features, the performance measures all decreased to different extents upon the removal of a particular feature. This implies that each variety of features in MULGA contributes non-negligibly to DPI prediction. The results also reveal the precise similarities among drugs and proteins since they harbor rich and complementary knowledge including molecular sequence, structural and physiochemical knowledge. However, different types of features may contribute to the performance to varying degrees. After removal of the PAAC features, the performance decreased compared to all features—a decrease of 3.79% in ACC and 5.10% in AUROC in the balanced scenario, and a decrease of 5.76% in ACC and 10.18% in AUROC in the imbalanced scenario, respectively, suggesting that PAAC is the most contributing feature (i.e. “view”) to the DPI prediction in MULGA.

### 3.4 “Guilty-by-association”-based negative sampling serves as an effective strategy for the selection of reliable negative DPIs

To evaluate the effectiveness of the proposed negative sampling strategy, we compared it with the random negative sampling scheme on both balanced and imbalanced data of DrugBank dataset. The results are shown in Fig. 4B, where dark blue cells represent higher performance and light blue cells denote lower scores. It is obvious from Fig. 4B that the proposed negative sampling method is superior to random sampling for MULGA. To intuitively explore the rationale behind this, we utilized the t-distributed stochastic neighbor embedding (*t*-SNE) (Hinton 2002) to visualize the extracted feature distribution of MULGA models trained with the two negative sampling strategies under the balanced scenario in Fig. 4C. The positive and negative DPI samples are labelled in green and red, respectively. As Fig. 4C shows, the model trained with our “guilty-by-association” scheme generates representations with more distinguishable and clearer feature distribution. Taken together, we conclude that our proposed “guilty-by-association” negative sampling scheme is a more advanced sampling strategy under the data imbalance conditions and significant for accurate DPI prediction.

### 3.5 “MULGA” is capable of predicting potentially effective drugs for targeting the spike glycoprotein of SARS-CoV-2

In this section, we further showcase a real-world application of MULGA to repositioning potential drugs for treating COVID-19, a global health crisis that caused massive mortality of over six million people worldwide (Huang *et al.* 2020). COVID-19 is caused by the severe acute respiratory syndrome coronavirus 2 (SARS-CoV-2), a single-stranded RNA-enveloped virus (Zhu *et al.* 2020). SARS-CoV-2 encodes various proteins, including spike glycoprotein, which is localized on the cell surface and plays an important role of mediating the viral cell entry (Huang *et al.* 2020). We applied MULGA to predict candidate drugs that can potentially target the spike glycoprotein. Particularly, we extracted the FASTA-format spike glycoprotein sequences of SAR-CoV-2 from the UniProt database (Bateman *et al.* 2019), followed by the calculation of the AAC, Moran autocorrelation, CTD, and PAAC features for these sequences. The target affinity matrix was then recalculated based on the newly added sequence. Meanwhile, the blank association vector of SAR-CoV-2 and other drugs was padded into the original drug–target association matrix. Finally, MULGA was implemented on the new heterogeneous drug–target matrix equipped with the recalculated target affinity and drug–target association matrices of DrugBank dataset. The top 20 candidate drugs for the spike glycoprotein were selected based on their predicted probability. Supplementary Fig. S5 provides the ranking of those top 20 drugs, and the supporting evidence can also be found in Supplementary File. Taken together, MULGA is capable of identifying and prioritizing potentially effective drugs to target the spike glycoprotein of SARS-CoV-2 can identify potential in identifying reliable DPIs for repurposing existing drugs for treating different diseases.

## 4 Discussion and conclusion

An advantage of MULGA is that it can incorporate multiple heterogeneous drug features and protein features irrespective of their size and dimension. Furthermore, it can also be extended to address other association prediction tasks, e.g. drug–drug interaction prediction, peptide–protein interaction prediction and protein–protein interaction prediction. However, MULGA also has limitations: first, although MULGA could unveil the binding situation among drugs and proteins, the specific binding sites of drugs and proteins still need to be characterized. In other words, investigating the binding sites making the most significant contribution toward the DPI prediction may be more relevant for the clinical trial; second, the pairwise “multi-view” learning method used in MULGA requires the features with equal length, posing challenges of incorporating versatile drug features and protein features; third, the species-specific knowledge was not incorporated into the MULGA framework when using the DrugBank and KIBA datasets. In the future work, we plan to develop new computational frameworks to address this issue by incorporating species-specific information and examining the possibility of transferring the knowledge learned by species-specific model to other diverse organisms, and fourth, the current version of MULGA lacks desirable biological interpretability to some extent. In this regard, we plan to improve this by integrating interpretable DL and/or attention-based mechanisms in future work.

## Supplementary Material

btad524_Supplementary_Data

## Data Availability

The DrugBank, KIBA, Davis, and BindingDB datasets and their processing pipelines are available at https://github.com/jianiM/MULGA. The source code of MULGA can also be found at https://github.com/jianiM/MULGA.
