# Supplementary material for: MULGA, a unified multi-view graph autoencoder-based approach for identifying drug–protein interaction and drug repositioning
Source: Bioinformatics. 2023 Aug 23;39(9):btad524. doi: 10.1093/bioinformatics/btad524 (PMC10518077; doi:10.1093/bioinformatics/btad524)
Supplement: btad524_Supplementary_Data [file btad524_supplementary_data.zip › Supplementary File_FINAL.docx]

**Supplementary File**

# MULGA, a unified multi-view graph autoencoder-based approach for identifying drug-protein interaction and drug repositioning

Jiani Ma1, Chen Li2, Yiwen Zhang3, Zhikang Wang2, Shanshan Li3, Yuming Guo3, Lin Zhang1, Hui Liu1,*, Xin Gao4,*, Jiangning Song2,5,6,*

1School of Information and Control Engineering, China University of Mining and Technology, Xuzhou, 221116, China; 2Monash Biomedicine Discovery Institute and Department of Biochemistry and Molecular Biology, Monash University, Melbourne, VIC 3800, Australia; 3Climate, Air Quality Research Unit, School of Public Health and Preventive Medicine, Monash University, Melbourne, VIC, 3004, Australia;4KAUST Computational Bioscience Research Center (CBRC), King Abdullah University of Science and Technology (KAUST), Thuwal 23955, Saudi Arabia; 5Wenzhou Medical University-Monash Biomedicine Discovery Institute (BDI) Alliance in Clinical and Experimental Biomedicine, Wenzhou, 325035, China; 6Monash Data Futures Institute, Monash University, Melbourne, VIC 3800, Australia.

*To whom correspondence should be addressed:

Jiangning Song, Biomedicine Discovery Institute and Department of Biochemistry and Molecular Biology, Monash University, Victoria 3800, Australia. Email: [jiangning.song@monash.edu](mailto:jiangning.song@monash.edu).

Xin Gao, Computational Bioscience Research Center, King Abdullah University of Science and Technology (KAUST), Thuwal 23955, Saudi Arabia. Email: [xin.gao@kaust.edu.sa](mailto:xin.gao@kaust.edu.sa).

Hui Liu, School of Information and Control Engineering, China University of Mining and Technology, Xuzhou, 221116, China. Email: [hui.liu@cumt.edu.cn](mailto:hui.liu@cumt.edu.cn).

**Supplemental methods**

1. **Feature-driven affinity matrix learning--updating rule for**

With all but one fixed (i.e., for views ***A***(1), ***A***(2),…, ***A***(*v*), we fixed *v*-1 views, and only took one view, ***A***(i), as a variable each time), the pairwise multi-view learning model can be separated as *v* independent minimization models, and the *i*-th pairwise multi-view learning model is defined as in (1):

Due to the inseparable property of variable , we translated the model (1) into its equivalent form (2) by introducing four auxiliary matrices , , , and . Then model (1) can be solved by applying the Alternating Direction Method of Multipliers (ADMM) framework(Eckstein, 2011) to model (2), and each variable can be optimized in an iterative scheme:

Accordingly, the augmented Lagrangian function is defined as (3):

where , ***Y***1(*i*), ***Y***2(*i*),***Y***3(*i*), ***Y***4(*i*) are Lagrange dual variables, and *μ* is the penalty parameter. At the *k*-th iteration, MULGA alternatively calculates ***A***1(*i*), ***A***2(*i*), ***A***3(*i*), ***A***4(*i*)­, ***Y***1(*i*), ***Y***2(*i*),***Y***3(*i*),and ***Y***4(*i*).

**The rule for updating *A*1(*i*) at the *k*-th iteration**: To obtain the optimal solution ***A***1(*i*)*, we minimized the augmented Lagrangian function (4) with respect to ***A***1(*i*):

where is the singular value thresholding operator(Cai, et al., 2010).

**The rule for updating *A*2(*i*) at the *k*-th iteration**: Given other variables in their (*k*-1)-th iteration, the optimal ***A***2(*i*) in the *k*-th iteration is:

where is the element-wise shrinkage operator(Donoho, 1995).

**The rule for updating *A*3(*i*) at the *k*-th iteration**: By minimizing (4) with respect to ***A*3(*i*),** the optimization of ***A***3(*i*) can be cast into the following task (6):

Setting the derivate of (6) with respect to ***A***3(*i*) to zero, we can achieve the closed-form solution of ***A***3(*i*)* as (7) shown below.

**The rule for updating *A*4(*i*) at the *k*-th iteration**: After fixing the other variables in their previous iteration, the optimal ***A***4(*i*) can be updated as follows:

The closed-form optimization of ***A***4(*i*) is performed by setting the derivate of (8) with respect to ***A***4(*i*) to zero:

**The rule for updating dual variables *Y***1(*i*), ***Y***2(*i*),***Y***3(*i*), ***Y***4(*i*) **at the *k*-th iteration**: Finally, the dual variables and penalty parameter μ can be updated by the following eq. (10):

These update steps should be repeated until the following convergence constraint is met:

Then, a series of affinity matrices of all views, i.e., ***A***(1), ***A***(2) , ***…*,*****A***(*v*), can be obtained, and the joint affinity matrix ***A*** is the element-wise average of these affinity matrices.

1. **Brief Introduction of Comparison Methods**

Numerical DTI computational models have been proposed in the recent two years. Most of them generally rely on diverse auxiliary information, including protein PDF structures, drug-side effect associations, drug-disease associations, even GO biological process, cellular components and molecular function information in DTI prediction task (Li, et al.; Li, et al., 2022; Liu, et al., 2022; Masumshah, et al., 2021; Peng, et al., 2021; Zhang, et al.) leveraged drug-side effect associations, drug-disease associations in DTI prediction. In the case of our proposed method MULGA, its inputs are protein fasta sequence and drug SMILES sequence. Based on the inputs, the drug fingerprint features and protein features can be extracted. As different methods use different inputs, it may not be fair to compare MULGA with the drug-disease association-based, drug-side effect information-based and PDB structure-based SOTA methods. Therefore, we chose the six sequence-derived computational methods, including DeepDTA(Ozturk, et al., 2018), DeepConv-DTI (Lee, et al., 2019), GraphDTA (Thin, et al., 2021), HyperAttentionDTI (Zhao, et al., 2022), MLMC (Yan, et al., 2022) and LRSpNM (Wu, et al., 2021) as the SOTA methods and compare their performance with that of MULGA. We trained and tested these methods based on the same 10-fold CV and same negative sampling scheme. A detailed description of each of the methods is provided below.

- **DeepDTA**

DeepDTA (Ozturk, et al., 2018)was initially proposed for predicting drug-protein affinity (DPA). DeepDTA encodes both drug SMILE sequence and protein fasta sequence using the 1D-CNNs, and then concatenates the extracted feature vectors of drugs and protein and feeds them into fully connected neural network (FCNN) to predict the drug-protein affinity. To fit for the DTI task, we applied a sigmoid function to the output layer, and thus cast the drug-protein affinity values to the probability of interaction.

- **DeepConv-DTI**

DeepConv-DTI (Lee, et al., 2019) applies FCNNs to encode drug fingerprints and uses 1D-CNN and global max-pooling layer to explore the sequence pattern of proteins. Then, DeepConv-DTI concatenates the extracted drug features and protein features and sends them to the fully connected neural network to generate the final prediction results.

- **GraphDTA**

GraphDTA (Thin, et al., 2021) was initially proposed for the DTA task. It combines GNNs and CNNs for drug structure and protein sequence representation, respectively. Similar to DeepDTA, we also added a sigmoid function in the last layer to transform it for the DTI task.

- **HyperAttentionDTI**

HyperAttentionDTI (Zhao, et al., 2022) is based on the attention mechanism. Taking drugs and proteins as sequences, HyperAttentionDTI utilized CNNs to learn the latent feature vectors of drugs and proteins. Then the attention blocks are applied on the extracted feature matrices to capture the local chemical interactions between drugs and proteins. After the max pooling layers, the modified drug-protein feature vectors are fed into fully connected neural networks to get the final DTI prediction.

- **MLMC**

MLMC (Yan, et al., 2022) is an ensemble method based on convex optimization strategy, which was initially proposed for drug-disease association prediction. Although the task is quite different from us, the ideas behind the methods are worthwhile for comparison. Thus, we rerun this method in the framework of DTI task. MLMC regards diverse information of drugs and diseases as different views, and uses an multi-view learning method with Laplacian graph regularization term to get comprehensive drug similarity matrix and protein similarity matrix. Finally, they uses the method bounded nuclear norm regularization (BNNR) method, which was proposed in (Yang, et al., 2019) predict DTIs with the aid of comprehensive drug similarity matrix and protein similarity matrix.

- **LRSpNM**

LRSpNM (Wu, et al., 2021) is a matrix completion method. Strictly, it is a non-convex optimization model rather than convex optimization model like MLMC. LRSpNM uses Schatten *p*-norm to approximate the matrix rank of DTI association matrix, and Laplacian regularization term to constrain the drug feature space and target feature space. Then it applies ADMM framework to decompose the optimization model, and uses GMST to solve the Schatten *p*-norm minimization sub-problem. After solving the non-convex minimization model, LRSpNM generates the complete low-rank DTI association matrix.

1. **Metrics**

(11)

(12)

(13)

(14)

(15)

where *TP*, *FP*, *TN*, *FN* denote the numbers of the true positive samples, false positive samples, true negative samples and false negative samples, respectively.

1. **Implementation and optimization**

We performed our experiments using PyTorch 1.11.0 with the Nvidia GeForce RTX 2080 GPU. Xavier initialization strategy was employed to the graph autoencoder to prevent gradient exploding and vanishing in the training phase. Next, we applied the Adam optimizer to train the graph autoencoder with the learning rate of 0.00001 and a dropout rate of 0.3, respectively. When training MULGA, we adopted the weighted cross entropy loss to reduce the influence of data imbalance and to focus on the validated DPIs:

(16)

where *y*+ and *y*- denote the positive and negative DPI instances, respectively, and |.| denotes the number of instances.

1. **Tuning** **hyperparameters of MULGA**

To further improve the performance of MULGA, we configured and optimized several parameters, including the trade-off weight *β*, penalty coefficient *μ*, the number of GCN layer *k,* and the hidden dimension of GCN layer *hgcn_dim*. More specifically, *β* and *μ* might have a great impact on the generation of the drug and protein affinity matrices, the input of the graph encoder. *k* is the maximum number of hops that each node can travel during the propagation process. *hgcn_dim* is not only the dimension of the hidden features but also the rank of ***H*R** and ***H*T**. Obviously, in MULGA, appropriate *β* and *μ* are the prerequisites for the downstream graph autoencoder pipeline. Therefore, we first performed grid search on *β* and *μ* with *k* and *hgcn_dim* fixed in the ‘warm-start’ scenario. To narrow down the search space, *β* and *μ* were empirically optimized from the set (0.1, 0.3, 0.5, 0.7, 0.9) and (21, 22, 23, 24), respectively.The value of *k* was chosen from (2, 3, 4) whilethe value of *hgcn_dim* was from (1000, 1500, 2000, 2500, 3000), respectively. With the element-wise average operator for achieving the joint drug affinity matrix and protein affinity matrix, the results of parameter tuning on the DrugBank dataset are presented in **Fig. S1.**

**
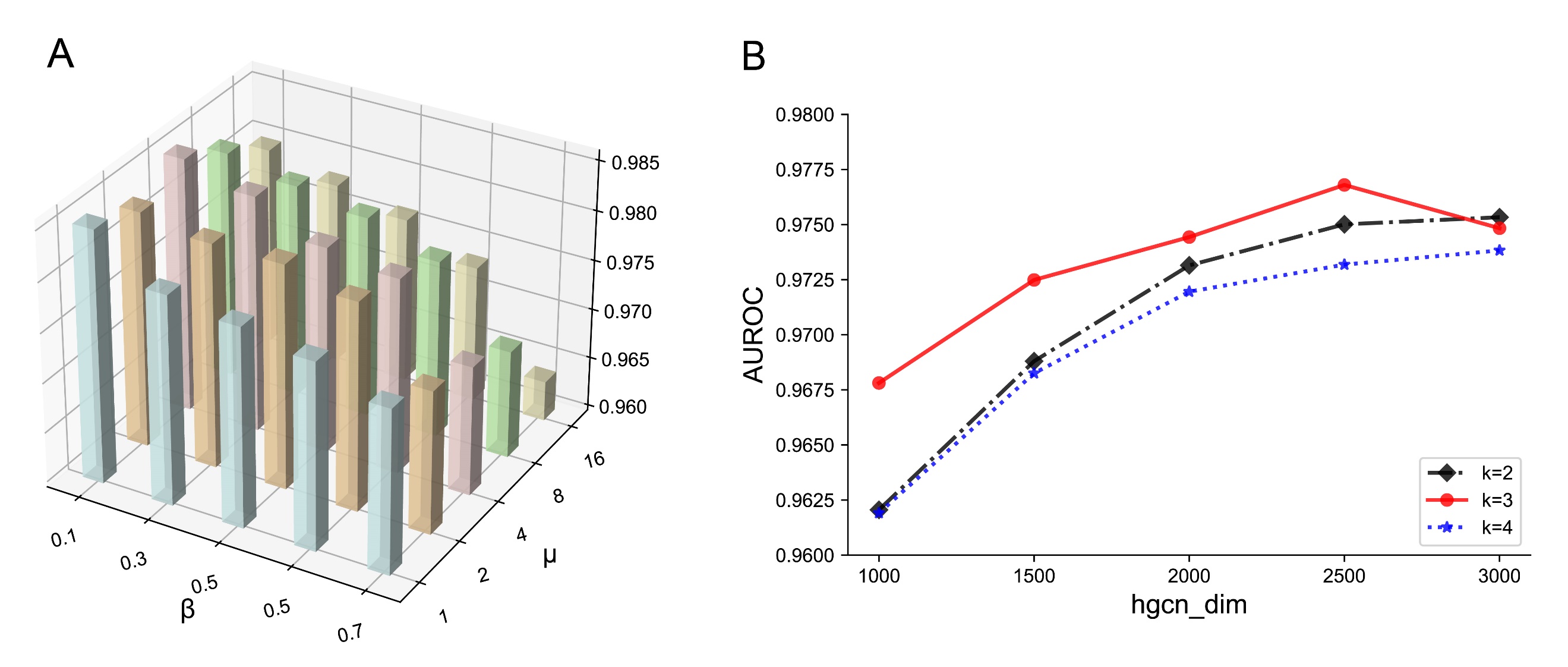
**

**Fig. S1.** The results of hyperparameter tuning for MULGA. **(A)** Grid search results of *β* and *μ* with fixed *k* and *hgcn_dim*. **(B)** Performance comparison of 2-layer GCN, 3-layer GCN and 4-layer GCN in terms of AUROC under different embedding dimensions.

**Fig. S1 (A)** shows changes of the AUROC scores with respect to the varying *μ* and *β*, with *k* = 3 and *hgcn_dim* = 2000. With a specific *μ* value, the AUROC scores increased as *β* decreased, following which MULGA achieved its highest AUROC score of 0.9755 with *μ* = 4 and *β* = 0.1. Then we further investigated the impact of *k* and *hgcn_dim* on the performance of MULGA. We set *k* as 2, 3, and 4 and performed the grid search based on the *hgcn_dim* values (**Fig. S1 (B**)). We can see that except for *k* = 3, the AUROC values increased as the value of *hgcn_dim* increased to 3000. Additionally, MULGA equipped with 3 GCN layers achieved a better performance than the counterparts with 2 and 4 GCN layers, indicating that the shallow embeddings may be more informative than the deeper stacking embeddings and over-smoothing of GCN can lead to poor performance (i.e., *hgcn_dim*=2500). Taken together, we set *μ* = 4, *β* =0.1, *k*=3, and *hgcn_dim*=2500 in MULGA for the DrugBank dataset in the follow-up experiments. In addition, we set *μ* = 4, *β* =0.5, *k*=3, and *hgcn_dim*=1800 in MULGA for the KIBA dataset, *μ* = 2, *β* =0.7, *k*=3, and *hgcn_dim*=200 for the Davis dataset and *μ* = 2, *β* =0.5, *k*=3, and *hgcn_dim*=1000 for the BindingDB dataset, respectively.

1. **Element-wise operator determination**

After the hyperparameter tuning procedure, we tested the influence of element-wise operators in prediction performance of MULGA, as they may influence the downstream GCN model and “guilty-by-association” negative sample scheme. To obtain the joint affinity matrix (e.g. drug affinity matrix and protein affinity matrix), we deployed three element-wise operators, including element-max, element-min and element-average, to all view-induced affinity matrices ***A***(1), ***A***(2),…, ***A***(v). Specifically, as for a specific pair aij,it can be calcuated by

(17)

Accordingly, we obtain ***A***(max), ***A***(min) and ***A***(average). Equipped with the downstream GCN model and “guilty-by-association” negative sampling scheme, we tested their overall performance under the balanced data setting on DrugBank, KIBA, Davis and BindingDB datasets. **Supplementary Table 1** showcases the mean and standard variance of AUROC and AUPR under balanced data setting with 10-fold CV on four datasets.

**Supplementary Table 1. Predictive performance of MULGA in terms of AUROC and AUPR on the balanced data setting using the four benchmark datasets.**

|  | **Element-wise operator** | **AUROC** |  | **AUPR** |
| --- | --- | --- | --- | --- |
| **DrugBank** | max | 0.9651 ± 0.0057 |  | 0.9599 ± 0.0060 |
|  | min | 0.9559 ± 0.0089 |  | 0.9620 ± 0.0061 |
|  | average | **0.9715 ± 0.0039** |  | **0.9743 ± 0.0038** |
| **KIBA** | max | 0.9400 ± 0.0076 |  | 0.9447 ± 0.0079 |
|  | min | 0.9331 ± 0.0064 |  | 0.9458 ± 0.0048 |
|  | average | **0.9479 ± 0.0035** |  | **0.9551 ± 0.0039** |
| **Davis** | max | 0.9399 ± 0.0084 |  | 0.9615 ± 0.0092 |
|  | min | 0.9320 ± 0.0121 |  | 0.9566 ± 0.0084 |
|  | average | **0.9405 ± 0.0080** |  | **0.9628 ± 0.0063** |
| **BindingDB** | max | 0.9832 ± 0.0089 |  | 0.9798 ± 0.0064 |
|  | min | 0.9776 ± 0.0114 |  | 0.9706 ± 0.0094 |
|  | average | **0.9874 ± 0.0093** |  | **0.9859 ± 0.0077** |

As shown in the **Supplementary Table 1**, element average operator exhibited consistent superiority with the highest mean AUROC and mean AUPR regardless of the datasets. View ***X***(1),  ***X***(2) ,*…* ,***X***(v) induce different but partial affinity information among samples, which are recorded in ***A***(1),  ***A***(2) ,*…* ,***A***(v). Furthermore, they captured consistent affinity information across different views and unique affinity information within the specific view. Thus, compared to simply taking the maximum affinity values or minimum affinity values across the views, taking the element-wise average appeared to be the best choice. This may be attributed to that the element-average operation did not change the consistent affinity information across different views and simultaneously it took all unique affinity information induced by each view into consideration.

1. **Comparison Results on KIBA**

**
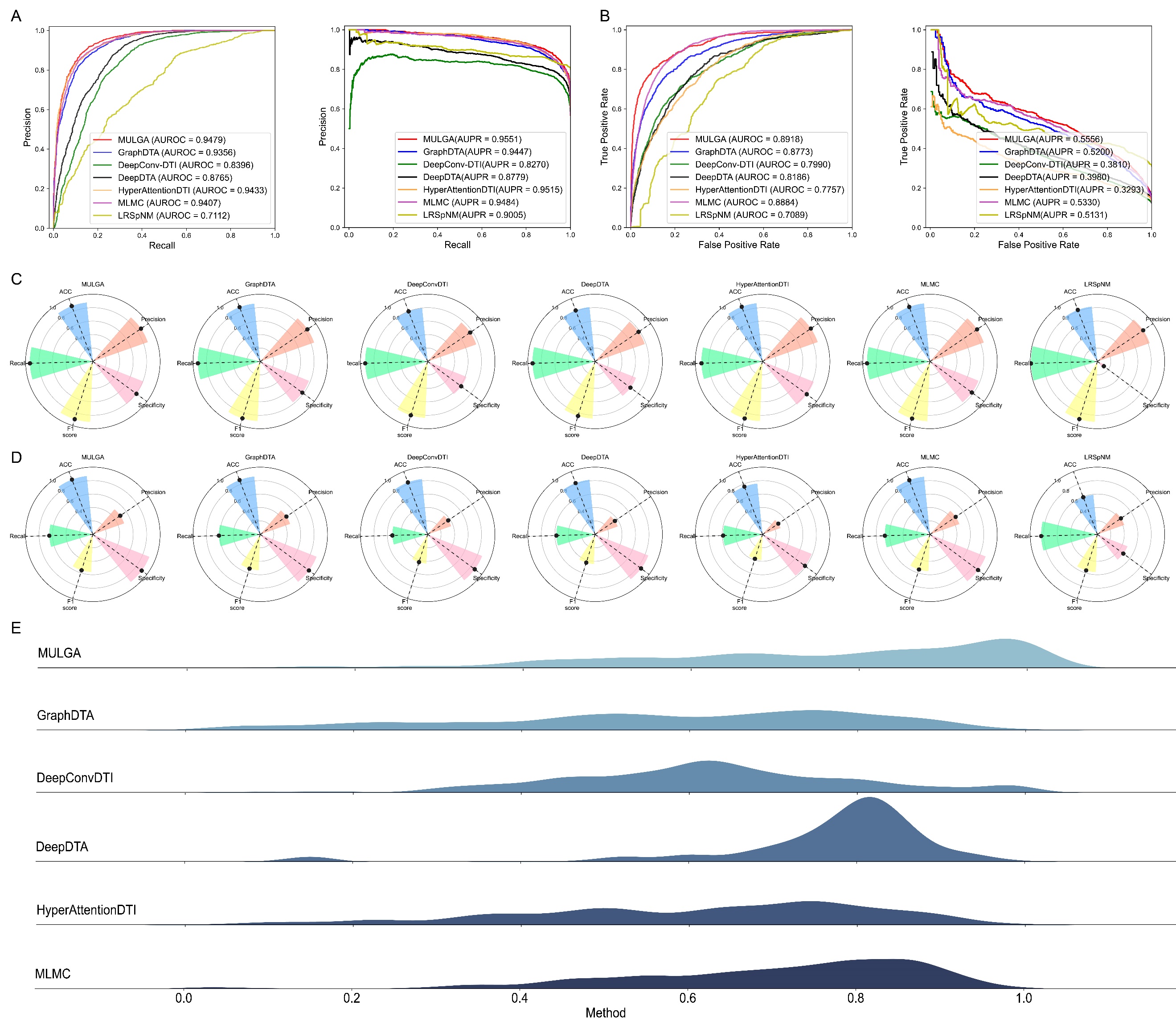
**

**Fig. S2.** Performance comparison of MULGA and other competing methods on the KIBA dataset. **(A)** ROC and PR curves of MULGA and its competing methods on the balanced dataset; **(B)** ROC and PR curves of MULGA and its competing methods on the imbalanced dataset; **(C)** Radar plots of MULGA and other methods on ACC, Precision, Recall, F1 score, and Specificity metrics on the balanced dataset; **(D)** Radar plots of MULGA and other methods on ACC, Precision, Recall, F1 score, and Specificity metrics on the imbalanced dataset, and **(E)** Performance comparison between MULGA and its competing methods under the “cold-start-for-protein” scenario.

**Fig. S2** shows the complete performance comparison results of MULGA with its competing methods on KIBA dataset. **Fig. S2A-B** show the AUROC and AUPR curves under balanced dataset and imbalanced dataset, respectively, where MUGLA has the best performance regardless of the datasets. **Fig. S2 C and D** illustrate the ACC, Precision, Specificity, Recall and F1 score of MULGA and its competing methods under balanced dataset and imbalanced dataset. MULGA still performs best in these indicators. Taking **Fig. S2A**-**D** into consideration, MLMC is the second-best method, which is slightly worse in those metrics than MULGA, which may contribute to the unbiased and extendable drug similarity matrix and target similarity matrix. In **Fig. S2E**. AUROC scores yielded right-skewed distributions ranging from 0.8 to 1.0, and MLMC exhibited a slightly right-skewed distributions with the AUROC scores concentrated within the interval [0.6,0.9]. In contrast, GraphDTA, DeepConv-DTI and HyperAttentionDTI exhibited normal distribution with the AUROC scores ranging from 0.4 to 0.8.

1. **Comparison Results on Davis**

**Fig. S3A-B** illustrate the ROC and PR curves of MULGA and its competing methods under the balanced data setting and imbalanced data setting, respectively. As can be seen, MULGA showed notable superiority with the highest mean AUROC score and highest mean AUPR score. Moreover, it still took a leading edge in terms of ACC, Precision, Recall, F1 score, and Specificity (shown in **Fig. S3C-D**). Under the “cold-start-for-protein” scenario, MULGA outperformed DeepConvDTI, DeepDTA, and MLMC with relatively right-skewed AUROC distributions. However, its distribution closely resembled to that of HyperAttentionDTI, both of which exhibited comparable performance (**Fig. S3E**).


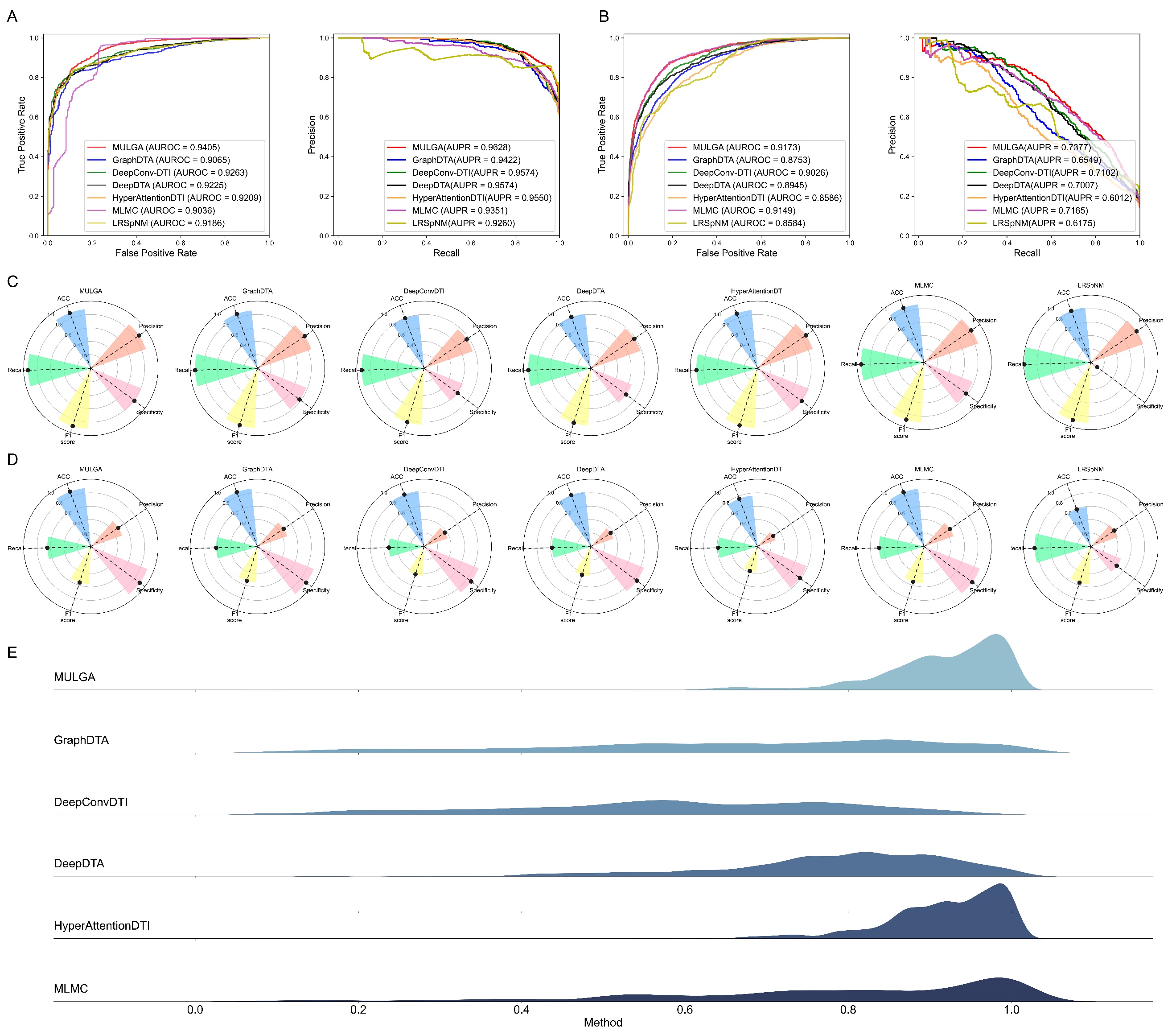


**Fig. S3.** Performance comparison of MULGA and other competing methods on the Davis dataset. **(A)** ROC and PR curves of MULGA and its competing methods on the balanced dataset; **(B)** ROC and PR curves of MULGA and its competing methods on the imbalanced dataset; **(C)** Radar plots of MULGA and other methods on ACC, Precision, Recall, F1 score, and Specificity metrics on the balanced dataset; **(D)** Radar plots of MULGA and other methods on ACC, Precision, Recall, F1 score, and Specificity metrics on the imbalanced dataset, and **(E)** Performance comparison between MULGA and the competing methods in the “cold-start-for-protein” scenario.

1. **Comparison Results on BindingDB**


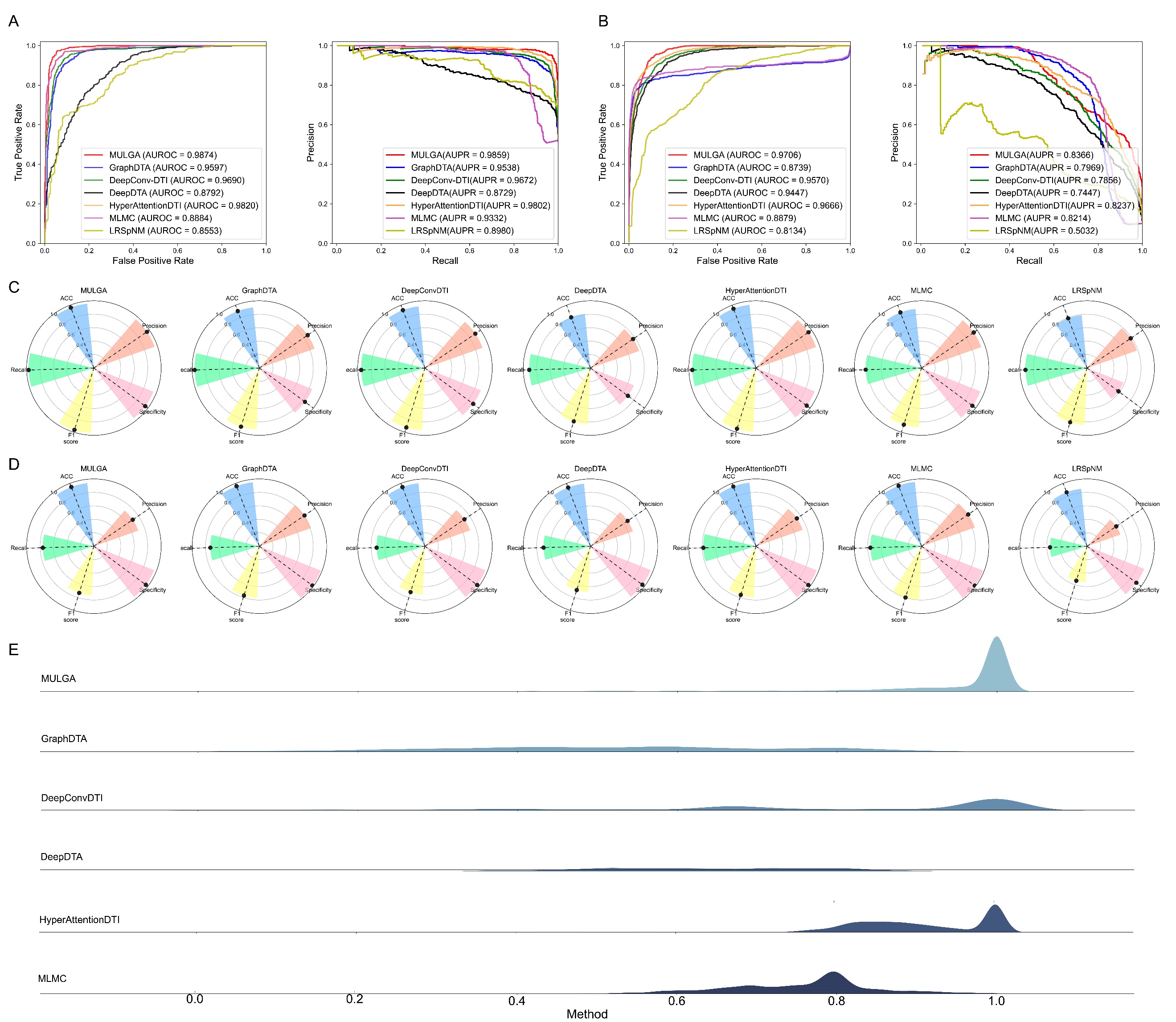


**Fig. S4.** Performance comparison of MULGA and other competing methods on BindingDB dataset. **(A)** ROC and PR curves of MULGA and its competing methods on the balanced dataset; **(B)** ROC and PR curves of MULGA and its competing methods on the imbalanced dataset; **(C)** Radar plots of MULGA and other methods on ACC, Precision, Recall, F1 score, and Specificity metrics on the balanced dataset; **(D)** Radar plots of MULGA and other methods on ACC, Precision, Recall, F1 score, and Specificity metrics on the imbalanced dataset, and **(E)** Performance comparison between MULGA and its competing methods under the “cold-start-for-protein” scenario.

**Fig. S4A-B** illustrate the overall performance of MULGA and its competing methods under both balanced data and imbalanced data settings. MULGA surpassed the second-best method HyperAttention by 0.54% in AUROC and 0.57% in AUPR under the balanced data setting, while by 0.40% in AUROC and 1.29% in AUPR under the imbalanced data setting, respectively. **Fig. S4C-D** also demonstrate its competitive performance in terms of other metrics. As shown in **Fig. S4E,** MULGA exhibited an extremely right-skewed distribution with the AUROC scores concentrated within the interval [0.9,1.0], establishing itself as the top-performing method under the “cold-start-for-protein” scenario.

1. **Prediction results and supporting findings of top20 ranked spike-related drugs**

**
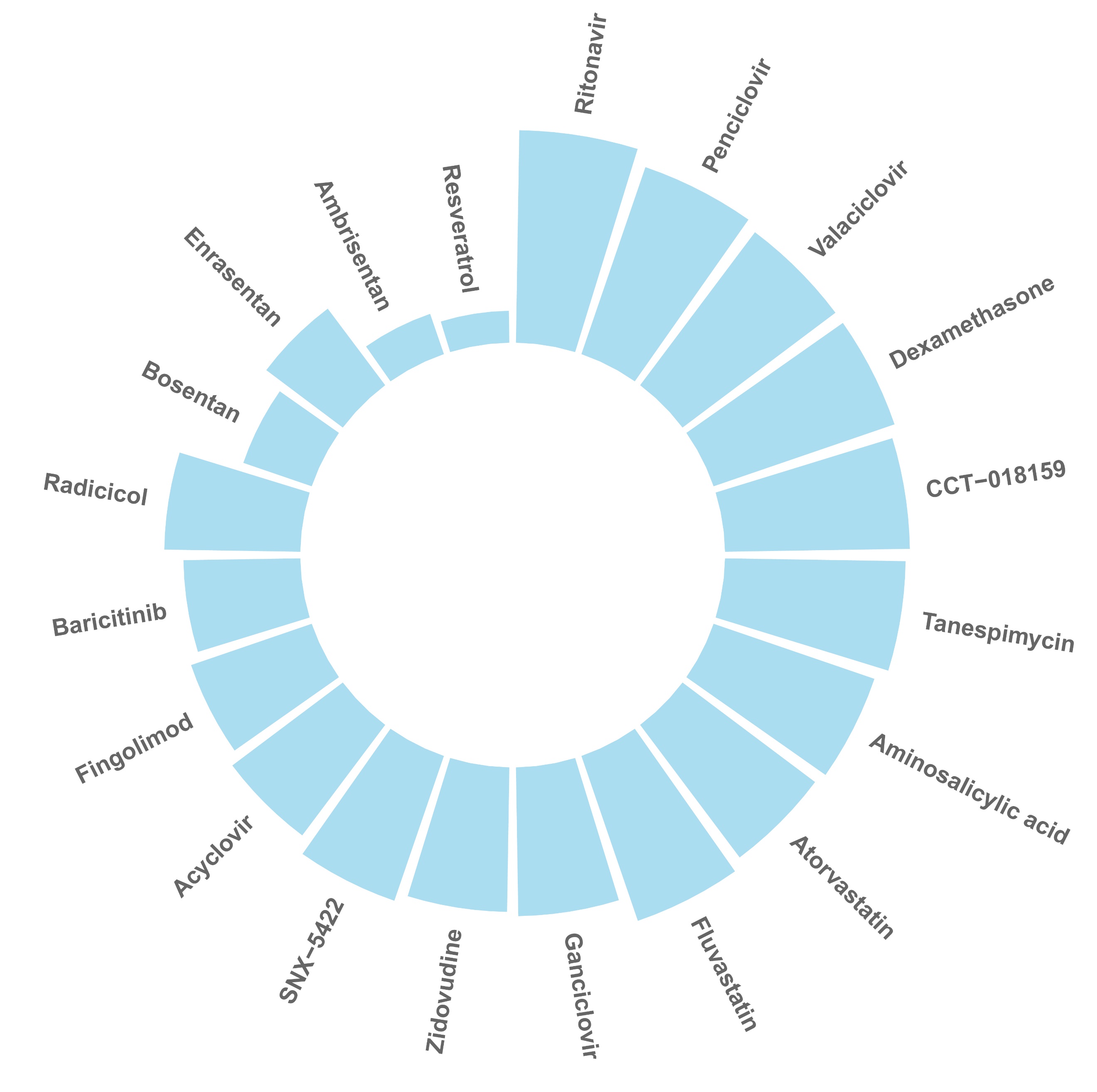
**

**Fig. S5.** Circular barplots of top20 ranked spike-related drugs predicted by MULGA. The higher bar denotes a higher probability whereas the lower bar denotes a lower probability.

Ritonavir and Dexamethasone were ranked as the top two predicted drugs that can potentially target the spike glycoprotein in **Fig. S5**. Together with the other two shortlisted drugs (i.e., Fingolimod and Baricitinib), these drugs have been proven experimentally effective but not approved for treating COVID-19(Patel, et al., 2021; Sullivan, et al., 2022). Despite that the effectiveness of Dexamethasone has been validated by the RECOVERY Collaborative Group(Horby, et al., 2021). For example, among the patients who received the invasive mechanical ventilation or oxygen alone, those who received the treatment of Dexamethasone have lower 28-day mortality(Horby, et al., 2021). Additionally, antiviral drugs, including Penciclovir, Valaciclovir, and Ganciclovir, were ranked as the third, fourth and fifth effective drugs, respectively. Recent clinical trials have pointed out that Penciclovir exhibits antiviral function against SARS-CoV-2 by binding to the nonstructural Protein 12 (nsp12) of SARS-CoV-2(Dey, et al., 2021), and Ganciclovir has also shown potential effectiveness in treating COVID-19(Yousefifard, et al., 2020).

**Supplementary Table 2. Performance results of view ablation study.**

|  | **Abandon View** | | **AUROC** | **AUPR** | **ACC** | **Precision** | **Recall** | **Specificity** | **F1 score** |
| --- | --- | --- | --- | --- | --- | --- | --- | --- | --- |
| Balanced dataset |  | \ | **0.9715**  **± 0.0039** | **0.9743**  **± 0.0038** | **0.9197**  **± 0.0059** | **0.9312**  **± 0.0176** | **0.9132**  **± 0.0132** | **0.9266**  **± 0.0211** | **0.9219**  **± 0.0051** |
|  | Drug fingerprints | MACCS key | 0.9672  ± 0.0046 | 0.9535  ± 0.0097 | 0.9092  ± 0.0065 | 0.9086  ± 0.0086 | 0.9112  ± 0.134 | 0.9072  ± 0.0218 | 0.9099  ± 0.0067 |
|  |  | Morgan | 0.9599  ± 0.0051 | 0.9561  ± 0.0065 | 0.9161  ± 0.0078 | 0.9196  ± 0.0135 | 0.9142  ± 0.0175 | 0.9180  ± 0.0209 | 0.9169  ± 0.0097 |
|  |  | Topological | 0.9535  ± 0.0039 | 0.9450  ± 0.0055 | 0.8979  ± 0.0093 | 0.9053  ± 0.0210 | 0.8908  ± 0.0234 | 0.9052  ± 0.0178 | 0.8980  ± 0.0102 |
|  | Protein features | AAC | 0.9554  ± 0.0049 | 0.9563  ± 0.0055 | 0.8906  ± 0.0067 | 0.8714  ± 0.0098 | 0.9170  ± 0.0154 | 0.8640  ± 0.0222 | 0.8936  ± 0.0123 |
|  |  | CTD | 0.9569  ± 0.0054 | 0.9546  ± 0.0062 | 0.8988  ± 0.0104 | 0.8839  ± 0.0188 | 0.9199  ± 0.0222 | 0.8774  ± 0.0287 | 0.9016  ± 0.0091 |
|  |  | Moran-Autocorrelation | 0.9540  ± 0.0057 | 0.9497  ± 0.0056 | 0.8891  ± 0.0069 | 0.8731  ± 0.0207 | 0.9112  ± 0.0265 | 0.8668  ± 0.0265 | 0.8917  ± 0.0085 |
|  |  | PAAC | 0.9206  ± 0.0111 | 0.9128  ± 0.0115 | 0.8819  ± 0.0186 | 0.8290  ± 0.0224 | 0.9665  ± 0.0199 | 0.7949  ± 0.0311 | 0.8925  ± 0.0155 |
| Imbalanced dataset |  | \ | **0.9259**  **± 0.0046** | **0.7179**  **± 0.0188** | **0.9407**  **± 0.0032** | **0.7128**  **± 0.0255** | **0.6548**  **± 0.0205** | **0.9715**  **± 0.0038** | **0.6822**  **± 0.0150** |
|  | Drug fingerprints | MACCS key | 0.9119  ± 0.0055 | 0.6014  ± 0.0162 | 0.9227  ± 0.0110 | 0.5850  ± 0.0185 | 0.5901  ± 0.0187 | 0.9569  ± 0.0231 | 0.5876  ± 0.0192 |
|  |  | Morgan | 0.9043  ± 0.0054 | 0.5522  ± 0.0167 | 0.9003  ± 0.0097 | 0.4832  ± 0.0176 | 0.6061  ± 0.0233 | 0.9314  ± 0.0188 | 0.5377  ± 0.0183 |
|  |  | Topological | 0.9183  ± 0.0077 | 0.6681  ± 0.0132 | 0.9330  ± 0.0129 | 0.6361  ± 0.0233 | 0.6463  ± 0.0200 | 0.9623  ± 0.0293 | 0.6412  ± 0.0144 |
|  | Protein features | AAC | 0.9108  ± 0.0100 | 0.6716  ± 0.0194 | 0.9349  ± 0.0187 | 0.6579  ± 0.0198 | 0.6186  ± 0.0212 | 0.9672  ± 0.0201 | 0.6377  ± 0.0194 |
|  |  | CTD | 0.9151  ± 0.0058 | 0.6708  ± 0.0212 | 0.9334  ± 0.0145 | 0.6488  ± 0.0283 | 0.6157  ± 0.0138 | 0.9659  ± 0.0233 | 0.6318  ± 0.0177 |
|  |  | Moran-Autocorrelation | 0.9127  ± 0.0058 | 0.6339  ± 0.0254 | 0.9307  ± 0.0243 | 0.6420  ± 0.0266 | 0.5691  ± 0.0276 | 0.9676  ± 0.0290 | 0.6034  ± 0.0215 |
|  |  | PAAC | 0.8250  ± 0.0088 | 0.4016  ± 0.0165 | 0.8800  ± 0.0178 | 0.3786  ± 0.0301 | 0.3814  ± 0.0289 | 0.9332  ± 0.0297 | 0.3800  ± 0.0245 |

**References**

Cai, J.-F., Candes, E.J. and Shen, Z. A SINGULAR VALUE THRESHOLDING ALGORITHM FOR MATRIX COMPLETION. *Siam Journal on Optimization* 2010;20(4):1956-1982.

Dey, S.K.*, et al.* Suramin, penciclovir, and anidulafungin exhibit potential in the treatment of COVID-19 via binding to nsp12 of SARS-CoV-2. *Journal of Biomolecular Structure & Dynamics* 2021.

Donoho, D.L. De-noising by soft-thresholding. *IEEE Transactions on Information Theory* 1995;41(3):613-627.

Eckstein, S.B.N.P.E.C.B.P.J. Distributed optimization and statistical learning via the alternating direction method of multipliers. *Foundations and Trends in Machine Learning* 2011;3(1):1-122.

Horby, P.*, et al.* Dexamethasone in Hospitalized Patients with Covid-19. *New England Journal of Medicine* 2021;384(8):693-704.

Lee, I., Keum, J. and Nam, H. DeepConv-DTI: Prediction of drug-target interactions via deep learning with convolution on protein sequences. *Plos Computational Biology* 2019;15(6).

Li, M.*, et al.* Metapath-aggregated heterogeneous graph neural network for drug-target interaction prediction. *Briefings in Bioinformatics*.

Li, Y.*, et al.* Drug-target interaction predication via multi-channel graph neural networks. *Briefings in Bioinformatics* 2022;23(1).

Liu, S.Y.*, et al.* Improved drug-target interaction prediction with intermolecular graph transformer. *Briefings in Bioinformatics* 2022;23(5).

Masumshah, R., Aghdam, R. and Eslahchi, C. A neural network-based method for polypharmacy side effects prediction. *Bmc Bioinformatics* 2021;22(1).

Ozturk, H., Ozgur, A. and Ozkirimli, E. DeepDTA: deep drug-target binding affinity prediction. *Bioinformatics* 2018;34(17):821-829.

Patel, T.K.*, et al.* Efficacy and safety of lopinavir-ritonavir in COVID-19: A systematic review of randomized controlled trials. *Journal of Infection and Public Health* 2021;14(6):740-748.

Peng, J.J.*, et al.* An end-to-end heterogeneous graph representation learning-based framework for drug-target interaction prediction. *Briefings in Bioinformatics* 2021;22(5).

Sullivan, R.*, et al.* COVID-19 Infection in Fingolimod- or Siponimod-Treated Patients: Case Series. *Neurology-Neuroimmunology & Neuroinflammation* 2022;9(1).

Thin, N.*, et al.* GraphDTA: predicting drug-target binding affinity with graph neural networks. *Bioinformatics* 2021;37(8):1140-1147.

Wu, G.*, et al.* De Novo Prediction of Drug-Target Interactions Using Laplacian Regularized Schatten p-Norm Minimization. *Journal of Computational Biology* 2021;28(7):660-673.

Wu, G.Y.*, et al.* De Novo Prediction of Drug-Target Interactions Using Laplacian Regularized Schatten p-Norm Minimization. *Journal of Computational Biology* 2021;28(7):660-673.

Yan, Y.X.*, et al.* Drug repositioning based on multi-view learning with matrix completion. *Briefings in Bioinformatics* 2022;23(3).

Yang, M.Y.*, et al.* Drug repositioning based on bounded nuclear norm regularization. *Bioinformatics* 2019;35(14):I455-I463.

Yousefifard, M.*, et al.* Antiviral therapy in management of COVID-19: a systematic review on current evidence. *Archives of academic emergency medicine* 2020;8(1):e45-e45.

Zhang, R.*, et al.* MHTAN-DTI: Metapath-based hierarchical transformer and attention network for drug-target interaction prediction. *Briefings in Bioinformatics*.

Zhao, Q.C.*, et al.* HyperAttentionDTI: improving drug-protein interaction prediction by sequence-based deep learning with attention mechanism. *Bioinformatics* 2022;38(3):655-662.
