# Supplementary material for: MULGA, a unified multi-view graph autoencoder-based approach for identifying drug–protein interaction and drug repositioning
Source: Bioinformatics. 2023 Aug 23;39(9):btad524. doi: 10.1093/bioinformatics/btad524 (PMC10518077; doi:10.1093/bioinformatics/btad524)
Supplement: btad524_Supplementary_Data [file btad524_supplementary_data.zip › Supplementary Table 1.docx]

**Supplementary Table 1. Predictive performance of MULGA in terms of AUROC and AUPR on the balanced data setting using the four benchmark datasets.**

|  | **Element-wise operator** | **AUROC** | **AUPR** |
| --- | --- | --- | --- |
| **DrugBank** | max | 0.9651 ± 0.0057 | 0.9599 ± 0.0060 |
|  | min | 0.9559 ± 0.0089 | 0.9620 ± 0.0061 |
|  | average | **0.9715 ± 0.0039** | **0.9743 ± 0.0038** |
| **KIBA** | max | 0.9400 ± 0.0076 | 0.9447 ± 0.0079 |
|  | min | 0.9331 ± 0.0064 | 0.9458 ± 0.0048 |
|  | average | **0.9479 ± 0.0035** | **0.9551 ± 0.0039** |
| **Davis** | max | 0.9399 ± 0.0084 | 0.9615 ± 0.0092 |
|  | min | 0.9320 ± 0.0121 | 0.9566 ± 0.0084 |
|  | average | **0.9405 ± 0.0080** | **0.9628 ± 0.0063** |
| **BindingDB** | max | 0.9832 ± 0.0089 | 0.9798 ± 0.0064 |
|  | min | 0.9776 ± 0.0114 | 0.9706 ± 0.0094 |
|  | average | **0.9874 ± 0.0093** | **0.9859 ± 0.0077** |
