# Supplementary material for: MULGA, a unified multi-view graph autoencoder-based approach for identifying drug–protein interaction and drug repositioning
Source: Bioinformatics. 2023 Aug 23;39(9):btad524. doi: 10.1093/bioinformatics/btad524 (PMC10518077; doi:10.1093/bioinformatics/btad524)
Supplement: btad524_Supplementary_Data [file btad524_supplementary_data.zip › Supplementary Table 2.docx]

**Supplementary Table 2. Performance results of view ablation study.**

|  | **Abandon View** | | **AUROC** | **AUPR** | **ACC** | **Precision** | **Recall** | **Specificity** | **F1 score** |
| --- | --- | --- | --- | --- | --- | --- | --- | --- | --- |
| Balanced dataset |  | \ | **0.9715**  **± 0.0039** | **0.9743**  **± 0.0038** | **0.9197**  **± 0.0059** | **0.9312**  **± 0.0176** | **0.9132**  **± 0.0132** | **0.9266**  **± 0.0211** | **0.9219**  **± 0.0051** |
|  | Drug fingerprints | MACCS key | 0.9672  ± 0.0046 | 0.9535  ± 0.0097 | 0.9092  ± 0.0065 | 0.9086  ± 0.0086 | 0.9112  ± 0.134 | 0.9072  ± 0.0218 | 0.9099  ± 0.0067 |
|  |  | Morgan | 0.9599  ± 0.0051 | 0.9561  ± 0.0065 | 0.9161  ± 0.0078 | 0.9196  ± 0.0135 | 0.9142  ± 0.0175 | 0.9180  ± 0.0209 | 0.9169  ± 0.0097 |
|  |  | Topological | 0.9535  ± 0.0039 | 0.9450  ± 0.0055 | 0.8979  ± 0.0093 | 0.9053  ± 0.0210 | 0.8908  ± 0.0234 | 0.9052  ± 0.0178 | 0.8980  ± 0.0102 |
|  | Protein features | AAC | 0.9554  ± 0.0049 | 0.9563  ± 0.0055 | 0.8906  ± 0.0067 | 0.8714  ± 0.0098 | 0.9170  ± 0.0154 | 0.8640  ± 0.0222 | 0.8936  ± 0.0123 |
|  |  | CTD | 0.9569  ± 0.0054 | 0.9546  ± 0.0062 | 0.8988  ± 0.0104 | 0.8839  ± 0.0188 | 0.9199  ± 0.0222 | 0.8774  ± 0.0287 | 0.9016  ± 0.0091 |
|  |  | Moran-Autocorrelation | 0.9540  ± 0.0057 | 0.9497  ± 0.0056 | 0.8891  ± 0.0069 | 0.8731  ± 0.0207 | 0.9112  ± 0.0265 | 0.8668  ± 0.0265 | 0.8917  ± 0.0085 |
|  |  | PAAC | 0.9206  ± 0.0111 | 0.9128  ± 0.0115 | 0.8819  ± 0.0186 | 0.8290  ± 0.0224 | 0.9665  ± 0.0199 | 0.7949  ± 0.0311 | 0.8925  ± 0.0155 |
| Imbalanced dataset |  | \ | **0.9259**  **± 0.0046** | **0.7179**  **± 0.0188** | **0.9407**  **± 0.0032** | **0.7128**  **± 0.0255** | **0.6548**  **± 0.0205** | **0.9715**  **± 0.0038** | **0.6822**  **± 0.0150** |
|  | Drug fingerprints | MACCS key | 0.9119  ± 0.0055 | 0.6014  ± 0.0162 | 0.9227  ± 0.0110 | 0.5850  ± 0.0185 | 0.5901  ± 0.0187 | 0.9569  ± 0.0231 | 0.5876  ± 0.0192 |
|  |  | Morgan | 0.9043  ± 0.0054 | 0.5522  ± 0.0167 | 0.9003  ± 0.0097 | 0.4832  ± 0.0176 | 0.6061  ± 0.0233 | 0.9314  ± 0.0188 | 0.5377  ± 0.0183 |
|  |  | Topological | 0.9183  ± 0.0077 | 0.6681  ± 0.0132 | 0.9330  ± 0.0129 | 0.6361  ± 0.0233 | 0.6463  ± 0.0200 | 0.9623  ± 0.0293 | 0.6412  ± 0.0144 |
|  | Protein features | AAC | 0.9108  ± 0.0100 | 0.6716  ± 0.0194 | 0.9349  ± 0.0187 | 0.6579  ± 0.0198 | 0.6186  ± 0.0212 | 0.9672  ± 0.0201 | 0.6377  ± 0.0194 |
|  |  | CTD | 0.9151  ± 0.0058 | 0.6708  ± 0.0212 | 0.9334  ± 0.0145 | 0.6488  ± 0.0283 | 0.6157  ± 0.0138 | 0.9659  ± 0.0233 | 0.6318  ± 0.0177 |
|  |  | Moran-Autocorrelation | 0.9127  ± 0.0058 | 0.6339  ± 0.0254 | 0.9307  ± 0.0243 | 0.6420  ± 0.0266 | 0.5691  ± 0.0276 | 0.9676  ± 0.0290 | 0.6034  ± 0.0215 |
|  |  | PAAC | 0.8250  ± 0.0088 | 0.4016  ± 0.0165 | 0.8800  ± 0.0178 | 0.3786  ± 0.0301 | 0.3814  ± 0.0289 | 0.9332  ± 0.0297 | 0.3800  ± 0.0245 |
