# Supplementary figures and images for: MULGA, a unified multi-view graph autoencoder-based approach for identifying drug–protein interaction and drug repositioning
Source: Bioinformatics. 2023 Aug 23;39(9):btad524. doi: 10.1093/bioinformatics/btad524 (PMC10518077; doi:10.1093/bioinformatics/btad524)

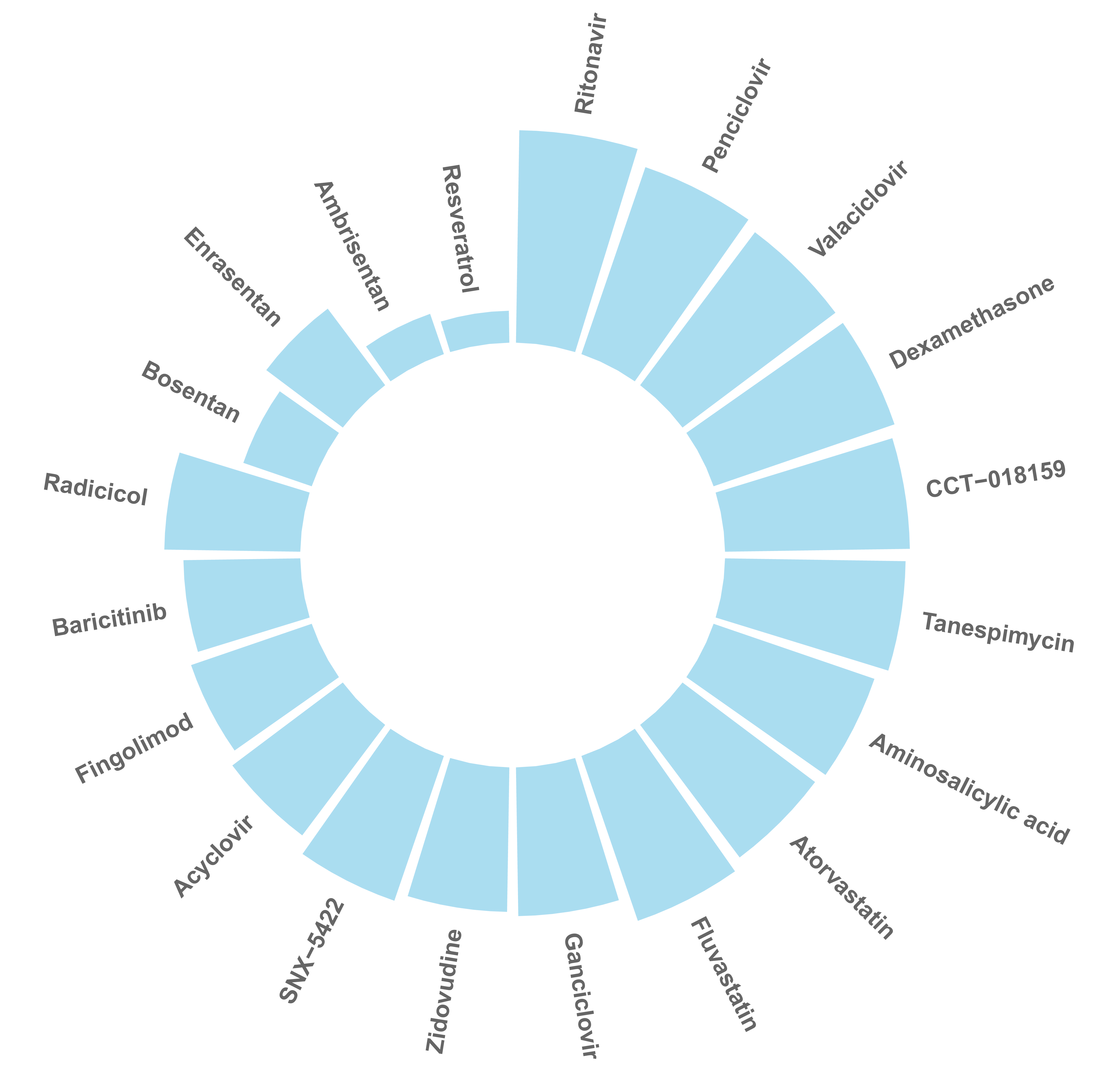

Supplement: btad524_Supplementary_Data [file btad524_supplementary_data.zip › figS5_revised.jpg]

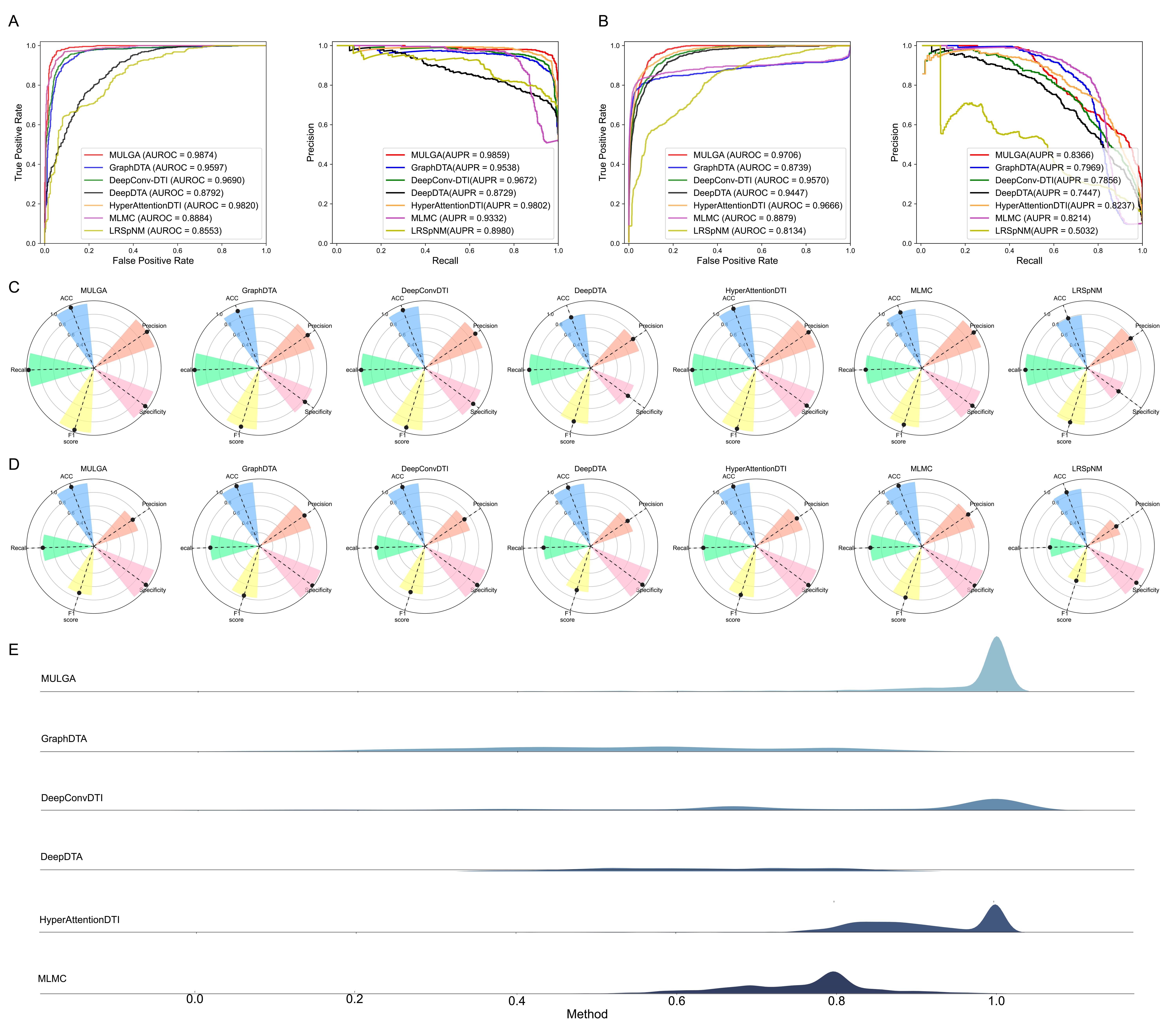

Supplement: btad524_Supplementary_Data [file btad524_supplementary_data.zip › figS4_revised.jpg]

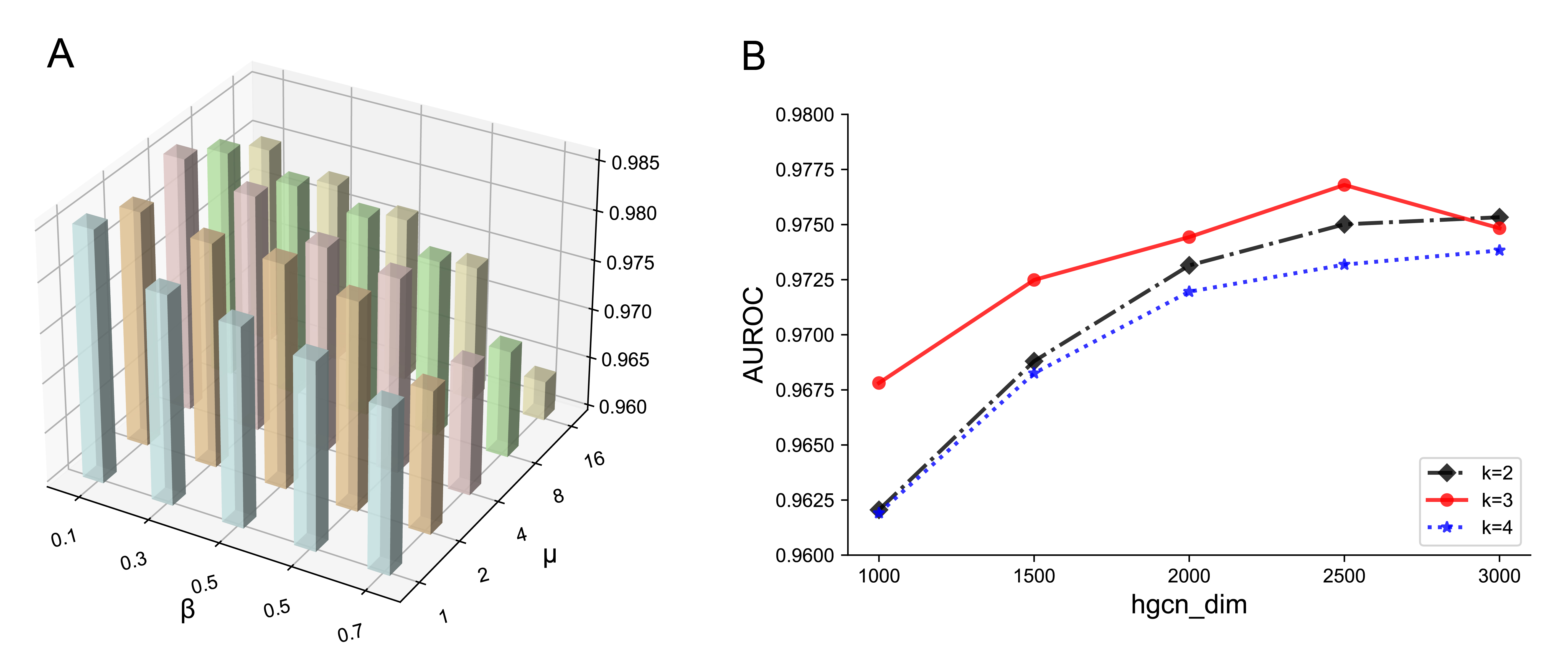

Supplement: btad524_Supplementary_Data [file btad524_supplementary_data.zip › figS1.jpg]

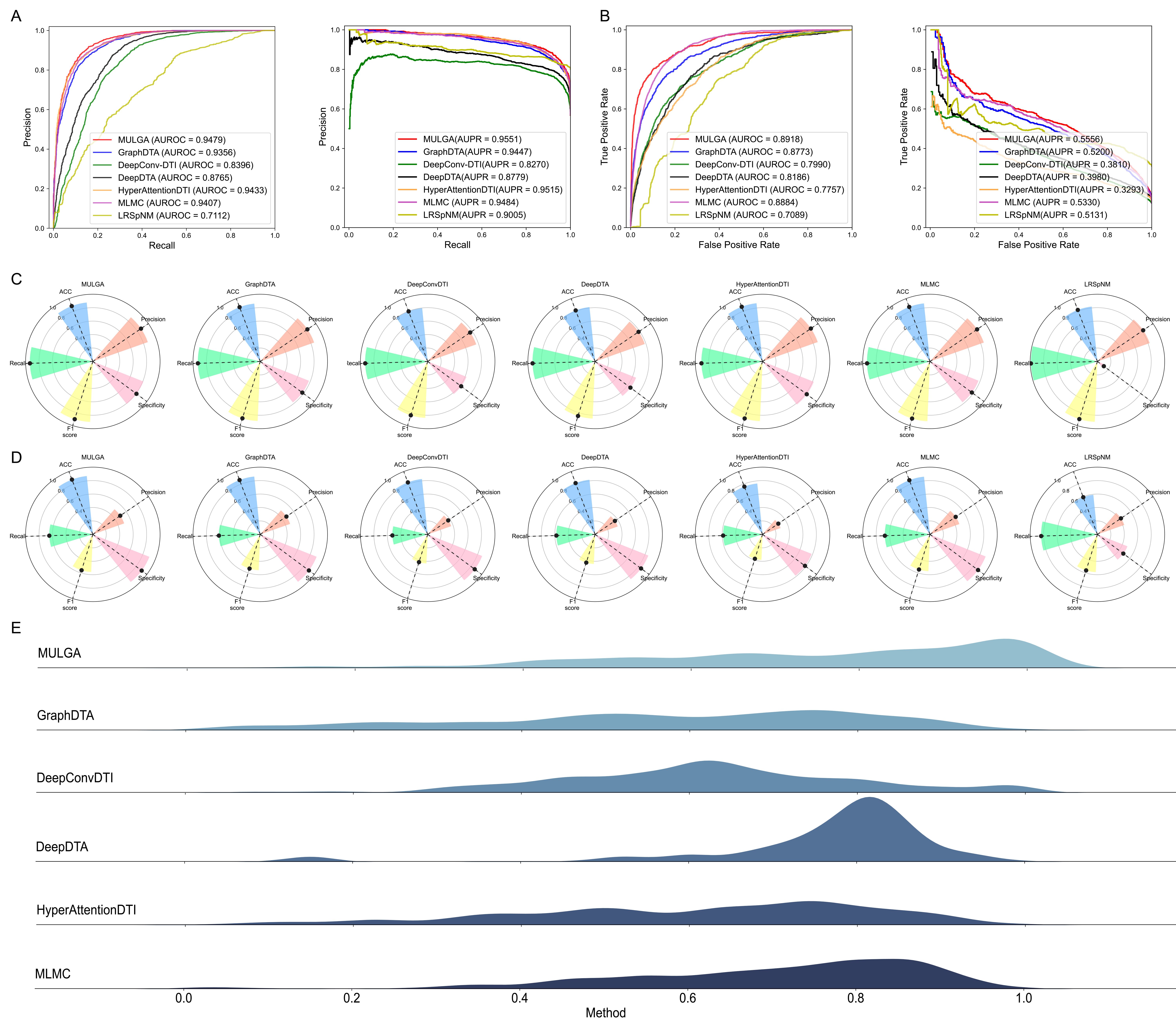

Supplement: btad524_Supplementary_Data [file btad524_supplementary_data.zip › figS2_revised.jpg]

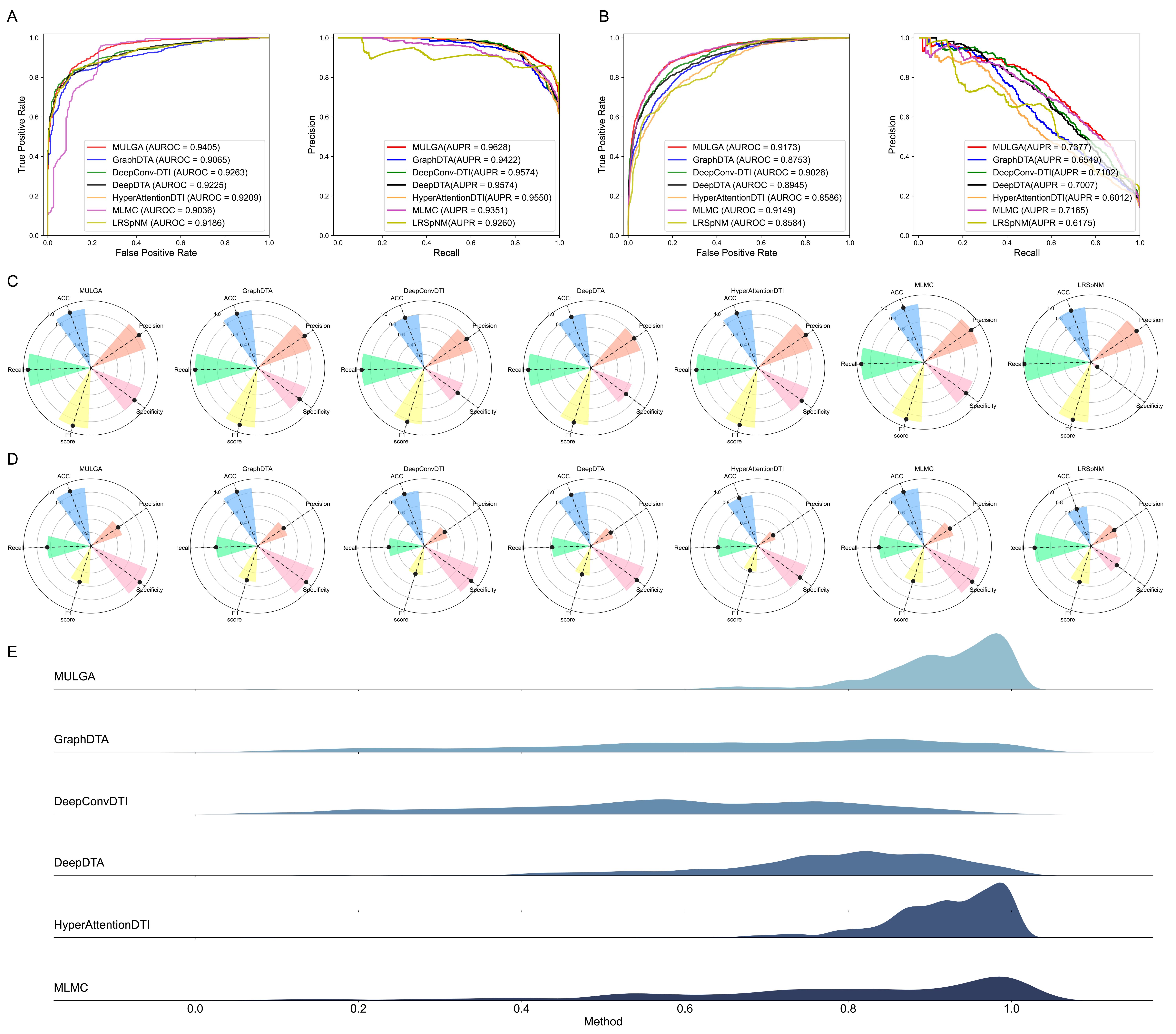

Supplement: btad524_Supplementary_Data [file btad524_supplementary_data.zip › figS3_revised.jpg]
